# Human *DDIT4L* intron retention contributes to cognitive impairment and amyloid plaque formation

**DOI:** 10.1038/s41421-024-00759-9

**Published:** 2025-02-11

**Authors:** Kai-Cheng Li, Hai-Xiang Shi, Zhen Li, Pu You, Jing Pan, Yi-Chuan Cai, Jin-Wen Li, Xue-Fei Ma, Shuo Zhang, Lei Diao, Bing Cai, Hai-Bo Wang, Liang Chen, Ying Mao, Xu Zhang

**Affiliations:** 1 QuietD Biotech, Shanghai, China; 2 Shanghai Advanced Research Institute, Chinese Academy of Sciences, Shanghai, China; 3 Huashan Hospital, Fudan University, Shanghai, China; 4 Present Address: Guangdong Institute of Intelligence Science and Technology, Zhuhai, Guangdong China

**Keywords:** Mechanisms of disease, Molecular biology

Dear Editor,

Current opinions about dementia, such as Alzheimer’s disease (AD), suggest that the aggregation of amyloid β (Aβ), followed by tau accumulation, occurs in the brain before the onset of cognitive defects, while ongoing efforts in suppressing Aβ formation or eliminating toxic Aβ have a modest clinical effect^1^. This suggests that there may be unidentified factors contributing to cognitive impairment in dementia. On the other hand, cerebral hypoxia is believed to be a risk factor for AD^2^. Hypoxia was reported to influence mRNA splicing^3^. Alternative splicing (AS) of pre-mRNA transcripts is a fundamental process that increases proteome diversity. One form of AS, intron retention (IR) of pre-mRNAs, may contribute to AD development^4,5^.

We reported that hypoxia induces aberrant splicing of human DNA damage-inducible transcript 4 like (*DDIT4L*), resulting in the production of an IR isoform (named DDIT4L intron retention (*DIR*)), which occurs in AD patients. The RNA level of *DIR* was significantly elevated in brain tissue of AD patients by IRfinder^6^ (Fig. 1a). The *DIR* mRNA level and DIR protein level increased in AD patients (Supplementary Fig. S1a–c). Furthermore, DIR had a high concentration in the blood and mainly colocalized with Aβ plaques in the hippocampus of AD patients (Fig. 1b, c).

**Fig. 1:**
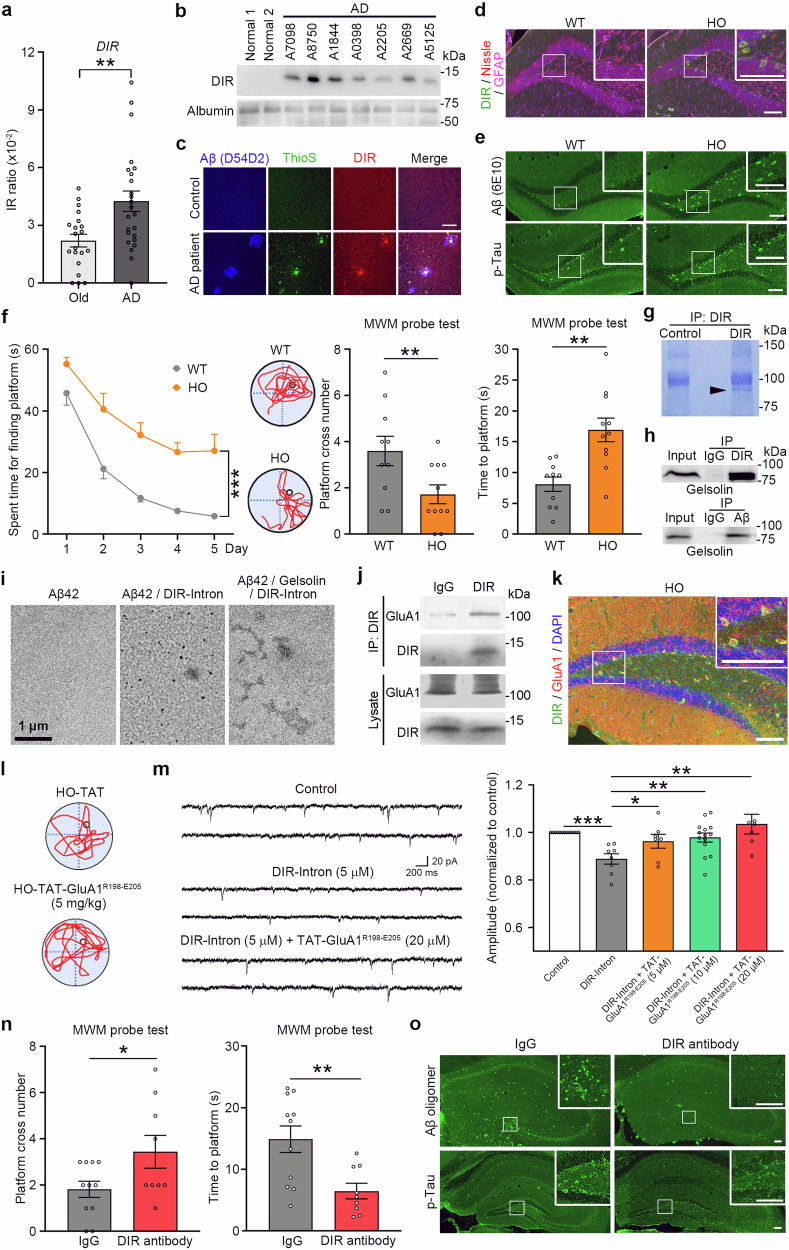
DIR binds to gelsolin, induces Aβ deposition, and interacts with GluA1 contributing to synaptic deficiency and cognitive impairment. **a** Increased expression of IR ratio of *DIR* from previous brain RNA-seq data of AD patients and old people (GSE153873 and GSE159699) using IRfinder. **P < 0.01. **b** The DIR was found in the AD patients’ plasma and individually named. **c** In AD patients, but not control individuals, DIR was distributed in the Aβ- and thioflavine S-positive dense-core plaques in the hippocampal DG area. Scale bar, 50 μm. **d** The DIR was expressed in the hippocampal DG neurons of 6-month-old HO mice. Scale bar, 100 μm. **e** The expression of Aβ (6E10) or p-Tau was upregulated in the hippocampal DG in the 6-month-old HO mice, compared with WT mice. Scale bars, 100 μm. **f** The Morris water maze test showed that HO mice increased the escape latency following a 5-day training period compared with that of WT mice. In the probe test to assess the spatial memory, WT mice spent more time crossing the platform and less time finding the platform compared with HO mice. **P < 0.01, ***P < 0.001. **g** Coomassie blue staining showed a band of molecular weight ∼85 kDa in the DIR-antibody-precipitated proteins from U87MG cells transfected with the Flag-DIR plasmid (*n* = 3). **h** In the lysate of HO hippocampi, gelsolin was found in the proteins precipitated with the DIR and Aβ antibodies. **i** Electron microscope showed Aβ42 (5 μM)/gelsolin (0.1 μM)/DIR-Intron (1 μM) incubation induced Aβ42 oligomerization. Scale bar, 1 μm. **j** In the hippocampus lysate from HO mice, GluA1 was found in the proteins precipitated with the DIR antibody. **k** The immunostaining showed that DIR was colocalized with GluA1 in HO mice. Scale bars, 50 μm. **l** Representative trial traces of individual HO mice treated with TAT or TAT-GluA1^R198–E205^ on probe test day. **m** sEPSC of hippocampal neurons was inhibited by the DIR-Intron (5 μM). TAT-GluA1^R198–E205^ reversed this inhibition in the brain slices prepared from WT mice. The amplitude of sEPSC in hippocampal neurons was suppressed by the DIR-Intron. TAT-GluA1^R198–E205^ could reverse this suppression in a dose-dependent manner (5, 10, 20 μM). *P < 0.05, **P < 0.01, ***P < 0.001. **n** In the probe test to assess spatial memory, HO mice treated with DIR mAb spent more time crossing the platform and less time finding the platform compared with the mice treated with IgG. *P < 0.05, **P < 0.01. **o** The expression of Aβ and p-Tau was decreased in the hippocampal DG in the 6-month-old HO mice treated with the DIR mAb. Scale bars, 100 μm. Data shown are mean ± S.E.M. Two-tailed unpaired *t*-test (**a**, **f**, **m**, **n**) or two-way ANOVA test followed by Bonferroni correction (**f**) was performed.

Next, we investigated the distribution and function of DIR by generating *DIR*-KI mice using CRISPR/Cas9 technology. The DDIT4L protein is conserved in many species (Supplementary Table 1). To induce the expression of human *DIR*, we inserted the sequence of the human *DIR* gene encoding the last 64 AAs after the mouse *DDIT4L* gene encoding AAs 1–20 (Supplementary Fig. S1d–g). An antibody against the IR portion of DIR showed the presence of one specific band (~12 kDa) in the hippocampi of homozygous *DIR*-KI mice (HO, Supplementary Fig. S1h–j). DIR was expressed in many hippocampal dentate gyrus (DG) neurons in HO mice (Fig. 1d; Supplementary Fig. S2a), some in the CA1 and CA3 areas, and a few cortical and thalamic neurons. The body weight of the HO mice was not significantly different from that of the wild-type (WT) mice, suggesting that the mutant mice had normal growth and metabolism. Moreover, the WT and HO mice showed no difference in distance travel in the open field test, indicating that motor function was not altered (Supplementary Fig. S2b, c).

DDIT4L could be upregulated by hypoxia in WT mice. The DIR protein expression was increased in neurons cultured under hypoxic conditions compared with control neurons (Supplementary Fig. S2d, e). Moreover, the expression of DIR was markedly enhanced in the hippocampi of HO mice exposed to low-oxygen conditions (8% O_2_) for 8 h (Supplementary Fig. S2f). Unilateral middle cerebral artery occlusion (MCAO) was used to mimic hypoxic-ischemic brain injury, and DIR expression was apparently increased in the ipsilateral hippocampi in HO mice subjected to MCAO but not in WT mice subjected to MCAO (Supplementary Fig. S2f, g). Thus, hypoxia could significantly enhance DIR expression in vivo.

To test whether the mouse intrinsic intron of *DDIT4L* could be retained, sequencing analysis was performed and showed that *mDIR* mRNA is transcribed in WT mice (Supplementary Fig. S3a, b). However, the *mDIR* could not be transcribed in HO mice and not be translated in the flag-mDIR transfect HEK293 cells and WT mice, while mDDIT4L expression was not changed in HO mice, as compared to that in WT mice in normal conditions (Supplementary Fig. S3b–e), suggesting that the DIR expression could be regulated by different spliceosome function in species.

The expression of Aβ and p-Tau was upregulated in the hippocampi of HO mice (Fig. 1e; Supplementary Fig. S4a–c), while DDIT4L expression showed no change in HO and WT mice (Supplementary Fig. S4d). Importantly, the DIR was partly co-localized with Aβ and p-Tau in hippocampal neurons of HO mice (Supplementary Fig. S5). To check the specificity of Aβ (6E10) and p-Tau antibodies, the signal of Aβ (6E10) and p-Tau antibody disappeared in the hippocampus of HO mice after the antigen absorption. Moreover, Aβ (6E10) was expressed in hippocampal neurons and accumulated at plaques deposited in the hippocampus of 5×FAD mice, while p-Tau could be detected in 3×Tg mice. (Supplementary Fig. S6a–d).

Next, we explored whether cognitive functions were impaired in HO mice. The results of the behavior test showed that cognition deficit occurred in the HO mice (Fig. 1f; Supplementary Fig. S7a, b and Videos S1, S2). To determine the functional domain of DIR, two polypeptides consisting of the first 30 AAs (DIR-Exon) or the last 54 AAs (DIR-Intron) of DIR were synthesized. Hippocampal slices from WT mice were incubated with either DIR-Exon, DIR-Intron, or DIR. Notably, DIR-Intron and DIR reduced the spontaneous excitatory postsynaptic current (sEPSC) amplitude and frequency, whereas neither the amplitude nor the frequency of sEPSCs was altered after DIR-Exon incubation (Supplementary Fig. S7c–g). Moreover, long-term potentiation (LTP) of field excitatory postsynaptic potentials (fEPSPs) recorded in the hippocampal CA1 region was decreased in the brain slices from HO mice compared with those from WT mice (Supplementary Fig. S7h). Furthermore, local delivery of the peptide DIR-Intron into the CA1 region of the hippocampus resulted in decreased novel object recognition (Supplementary Fig. S7i).

Next, we tested to know whether the mouse intrinsic intron of *DDIT4L* contributes to learning and memory. The specific siRNA for *mDIR* did not affect the behavior performance in WT or *APP/PS1* mice (Supplementary Fig. S8a–f). In AD patient samples, DIR mainly colocalized with Aβ in the hippocampus (Supplementary Figs. S8g, S9a, b). The interaction between DIR and Aβ was observed in transfected HEK293 cells, but DDIT4L (including DIR-Exon) could not interact with Aβ. Proximity ligation assays showed that DIR did not directly interact with Aβ. Synthetic DIR-Intron could not directly bind to Aβ (Supplementary Fig. S9c–f), suggesting that DIR has another binding target. Then DIR was transfected into human brain cell lines, and the cell lysates were immunoprecipitated with an anti-DIR antibody, and one immunoreactive band with a molecular weight of ~85 kDa was coimmunoprecipitated (Fig. 1g). The band was extracted and further analyzed by mass spectrometry, which revealed 12–15 peptides that matched the human gelsolin protein and covered 35% of the gelsolin sequence.

Gelsolin has been shown to interact with Aβ^7^, forming a complex that is transported to the circulatory system^8^. Our study showed that DIR interacted with gelsolin (Fig. 1h; Supplementary Fig. S9g–i). In the lysates of HEK293 cells that were transfected with DIR and endogenously expressed gelsolin, the additional Aβ42 enhanced the interaction of DIR with gelsolin in a dose-dependent manner, indicating that Aβ contributes to the interaction between DIR and gelsolin (Supplementary Fig. S10a). Purified gelsolin directly interacted with DIR (Supplementary Fig. S10b). A possible docking site for DIR in gelsolin was checked by a combination of homology modeling and in-silico docking^9^. The C228–N233 motifs in gelsolin (Gelsolin^C228–N233^) are possible docking sites for DIR. The interaction between DIR and gelsolin could be disturbed by the synthesized TAT-Gelsolin^C228–N233^ peptide. Moreover, C228 in gelsolin is the key site for the interaction between DIR and gelsolin (Supplementary Fig. S10c–f). Interestingly, TAT-Gelsolin^C228–N233^ peptide improved cognitive performance in HO mice, but not in *APP/PS1* mice in a dose-dependent manner (Supplementary Fig. S11a–c).

Moreover, the synthetic DIR-Intron and Aβ42 proteins were added to the purified gelsolin protein or HEK293 cell lysates, DIR-Intron enhanced the formation of insoluble Aβ42 including both Aβ42 monomers and oligomers, which is a major constituent of amyloid plaques (Fig. 1i; Supplementary Figs. S12a, b, S13a). The thioflavine T fluorescence assay showed that DIR-Intron bound gelsolin induced Aβ plaque in a short time (Supplementary Fig. S13b). Therefore, the intron region of DIR is involved in Aβ deposition.

However, thioflavine S did not label the amyloid plaques in the mouse hippocampi, because the murine Aβ sequence cannot be detected by thioflavine S^10^. Interestingly, the slices of hippocampus from HO mice that were incubated with human Aβ42 or Aβ40 could be stained by thioflavine S (Supplementary Figs. S13c, d, S14a), which was consistent with the thioflavine S staining of human Aβ plaques. Importantly, DIR was colocalized with gelsolin and Aβ to a greater degree in dense-core plaques than in diffuse plaques in AD patients, while no obvious DIR or amyloid plaque was stained in control subjects without AD (Supplementary Figs. S13d, S14b, c). However, DIR had not been involved in APP processing/Aβ production, as well as Aβ degradation (Supplementary Fig. S14d). Therefore, DIR may be an important molecule for Aβ deposition and plaque formation.

Then, we further explored whether there was a new target for DIR in AD. The hippocampal lysates of HO mice were immunoprecipitated with an anti-DIR antibody. One immunoreactive band with a molecular weight of ~110 kDa was coimmunoprecipitated. The band was extracted and further analyzed by mass spectrometry. This analysis identified 13 peptides that matched the human GluA1 protein, covering 14% of the GluA1 sequence (Supplementary Fig. S15a, b). Immunoprecipitation results showed that DIR interacted with GluA1 co-expressed with DIR in the hippocampal neurons of HO mice (Fig. 1j, k; Supplementary Fig. S15c–e), and purified GluA1 and DIR directly interacted (Supplementary Fig. S15f). We investigated the key motifs that were involved in the DIR modulation of AMPA receptor activation. A possible docking site for DIR in GluA1 was checked by a combination of homology modeling and in silico docking^9,11^. The R198–E205 motifs in GluA1 (GluA1^R198–E205^) were the possible docking site for DIR (Supplementary Fig. S15g). The interaction between DIR and GluA1 could be disturbed by the synthesized TAT-GluA1^R198–E205^ peptide (Supplementary Fig. S15h, i). Moreover, C204 in GluA1 was identified as the key site for the interaction between DIR and GluA1 (Supplementary Fig. S15j).

TAT-GluA1^R198–E205^ or control peptide intraperitoneally injected into HO mice crossed the blood–brain barrier (BBB, Supplementary Fig. S16a), and reversed cognitive impairment in HO mice, but not in *APP/PS1* mice (Fig. 1l; Supplementary Fig. S16b, c). The inhibitory effect of DIR-Intron on sEPSCs could be reversed by TAT-GluA1^R198–E205^ (Fig. 1m). The decrease in hippocampal LTP in the HO mice could be rescued by TAT-GluA1^R198–E205^ (Supplementary Fig. S16d). However, TAT-GluA1^R198–E205^ treatment did not change the expression of Aβ and p-Tau in the hippocampi of HO mice (Supplementary Fig. S16e).

To examine whether mAb against DIR could alleviate AD-like pathogenesis, DIR mAb or IgG was injected into HO mice. Cognitive impairment was improved by mAb treatment (Fig. 1n; Supplementary Fig. S17a, b and Videos S3, S4). The mAb could be detected in brain tissues by ELISA (Supplementary Fig. S17c), and DIR mAb-FITC could be diffused in the brain tissue (Supplementary Fig. S17d), suggesting DIR mAb crossing the BBB into brain tissues. The interaction between DIR and GluA1 or DIR and gelsolin was reduced in the hippocampi of HO mice after DIR mAb treatment (Supplementary Fig. S17e, f). Moreover, DIR mAb treatment reduced expression of Aβ and p-Tau (Fig. 1o; Supplementary Fig. S17g). The decrease in hippocampal LTP in HO mice could be rescued by the mAb (Supplementary Fig. S17h). Hippocampal slices from HO mice intraperitoneally injected with DIR mAb showed increased sEPSC amplitude, compared with those injected with IgG (Supplementary Fig. S17i).

The presence of a new pathogenic molecule, DIR, was identified in AD patients. Hypoxia could enhance the DIR expression. The human DIR*-*carrying mice exhibited accumulation of Aβ and p-Tau in the hippocampus as well as cognitive impairment, which correlated with a suppressive effect of DIR on hippocampal LTP. Furthermore, inhibition of DIR function in the HO mice alleviated cognitive decline, suggesting the potential of DIR as a target for dementia therapies.

Cerebrovascular sclerosis and other pathological conditions that lead to hypoxia often occur in elderly individuals^2,12^. The subsequent hypoxia in local microcerebrovascular circulation may result in abnormal IR^3,4^, including the translation of the DIR protein. Then, DIR bound to gelsolin and induced Aβ deposition to form dense-core plaques in the brain. Moreover, DIR interacted with GluA1, contributing to synaptic deficiency and cognitive impairment. Therefore, DIR would be a novel potential therapeutic target for dementia.

## Supplementary information

Supplementary information

Supplementary video S1

Supplementary video S2

Supplementary video S3

Supplementary video S4
