# Supplementary material for: Human DDIT4L intron retention contributes to cognitive impairment and amyloid plaque formation
Source: Cell Discov. 2025 Feb 11;11:12. doi: 10.1038/s41421-024-00759-9 (PMC11811001; doi:10.1038/s41421-024-00759-9)
Supplement: Supplementary file 1 — Supplementary information [file 41421_2024_759_MOESM1_ESM.docx]

**Supplementary information**

**Materials and methods**

**Human samples**

The human brain samples collection was conducted with the approval of the Ethics Committee of Huashan Hospital of Fudan University. Informed consent was obtained from all subjects involved in this study. The plasma of control and AD patients were used to examine the DIR. The information of enrolled patient was supplied in Supplementary table 2.

**Homozygous DIR-KI mice**

All animal operations were under the guidelines of the National Center for Protein Science Shanghai. Mice were accommodated in the specific pathogen-free rooms and supplied with enough foods and water. The DIR-KI mice were generated by Shanghai Model Organisms Center, Inc. Briefly, a cDNA encoding human *DIR* was inserted into mouse *DDIT4L* gene locus (Supplementary Fig. 1d, e) through homologous recombination by using CRISPR/Cas9 technology. All mice were maintained on a C57BL/6J background. The neonatal mice were identified by the PCR analysis, using the following genotyping primers.

Forward primer: 5’-GAGGAGCCTGTGCACTTCTT-3’;

Reverse primer: 5’-CACACCCAGCCTGTACACTT-3’.

The adult mice were anaesthetized and sacrificed. Then the brain tissues were isolated and homogenized. Next, total mRNAs were extracted using Trizol regents and 1 μg mRNAs were transcribed reversely to cDNA.

The following primers were used to detect the expression of DIR mRNA.

Forward primer: 5’-TGCCTCGGTTTACCCTTC-3’;

Reverse primer: 5’-AAGATGTTAGAAAATTTGGAAAGG-3’.

The following primers were used to detect the expression of mDIR mRNA.

Forward primer: 5’-ACGGGCAGTTTGAGCAGTAA-3’;

Reverse primer: 5’-CACAAGTAAGCGCATGTCTTT-3’.

The following primers were used to detect the expression of mDDIT4L mRNA.

Forward primer: 5’-TGCCTCGGTTTACCCTTC-3’;

Reverse primer: 5’-CCAGCTTTTTACATACATTTTCA-3’.

The following primers were used to detect the expression of GAPDH mRNA.

Forward primer: 5’-TATGTCGTGGAGTCTACTGGTGTCTTCACC-3’;

Reverse primer: 5’-GTTGTCATATTTCTCGTGGTTCACACCC-3’.

**Antibodies**

The DDIT4L and DIR antibodies used in our research were produced by GL Biochem (Shanghai) Ltd. The polyclonal antibodies used for Western blotting were generated from the rabbits, while the monoclonal antibodies for immunostaining and intraperitoneal injection were generated from the hybridoma.

To test the specificity of antibodies, the antibodies and the antibodies pre-absorbed with the antigens (10^-5^ or 10^-6^ M) for 12 h at 4°C were used to incubate the nitrocellulose membranes containing the same lysate samples, respectively. The nitrocellulose membranes were then incubated with HRP ligated second antibodies and imaged. Moreover, the antibody specificity also tested with the immunostaining of brain sections of homozygous DIR-KI mice.

**Immunoprecipitation**

The HEK293 cells and brain tissue of DIR-KI mice were lysed in the ice-cold buffer (25 mM Tris, 150 mM NaCl, 1 mM EDTA, 1% NP40, 5% glycerol and protease inhibitors, PH = 7.5). The suspended lysate was immunoprecipitated with the antibodies overnight at 4°C and then added Protein G-Agarose to bind the antibodies for 4 h at 4°C. The sepharose was then resuspended in the RIPA buffer without SDS, washed at least 3 times, and incubated in the SDS buffer for 20 min at 60°C. Then the immunoblotting was processed. The immunoblot band in DIR-KI mice was analyzed with the mass spectrometry.

**Immunoblotting**

Adult mice were anaesthetized and perfused with the PBS. Then brain tissues were extracted and homogenized on ice in the RIPA buffer (100 mM Tris, 150 mM NaCl, 1% Triton X-100, 10% glycerol, 0.05% BSA, 0.1% SDS and protease inhibitors, PH = 7.5). After centrifuged, supernatants of brain lysates were collected for further experiments. The brain tissues of human were lysed with the same tissue RIPA buffer.

The plasma of human was first diluted using the PBS at a ratio 1:10, about 10 μl samples of each diluted plasma were loaded to gels. All samples were fractionated by the sodium dodecyl sulfate (SDS)-gel electrophoresis, followed by being transferred onto the nitrocellulose filter membranes, and blocked with 5% nonfat milk in the TBS buffer containing Tween-20 (TBST). The membranes were incubated in the primary antibodies overnight at 4°C. Next day, membranes were washed with the TBST for three times and incubated with the HRP ligated second antibodies. After additional washing, the membranes were prepared to be imaged by adding the ECL buffer. Protein bands were imaged by using an imaging system (GE).

The primary antibodies used were listed here: Flag (Sigma, SAB4200071), Myc (Cell Signaling Technology, 2276S), Actin (Chemicon, MAB1501), Tubulin (Sigma, T5168), GAPDH (Proteintech, 10494), Gelsolin (Abcam, ab109014), GluA1 (Synaptic Systems, 182011), Aβ (Cell Signaling Technology, 8243S), and GFP (Invitrogen, A11122).

**Immunohistochemistry**

Briefly, mice were anaesthetized and perfused with Lana’s fix buffer or 4% PFA. Then brain tissues were collected and fixed in new fix buffer at 4°C for 1 h. After washing in PBS for three times, tissues were placed in 30% sucrose/PBS overnight at 4°C, then mounted in the OCT compound. Frozen section was performed to obtain brain section (40 µm in thickness). For immunostaining, tissue sections were blocked with PBST (0.1% Triton X-100/2.5% normal donkey serum/PBS) for 30 min at room temperature, followed by the incubation with primary antibodies overnight at 4°C. Then tissue sections were washed with PBS for three times and incubated with Alexa fluorescence-conjugated secondary antibodies (Invitrogen) for 60 min at 37°C, then washed with PBS three times and mounted with medium containing DAPI. The images were acquired using a Leica SP8 confocal microscope. The primary antibodies used were listed here: GFAP (Sigma, G9269), Aβ (BioLegend, 803001), Aβ oligomer (Invitrogen, AHB0052), p-Tau (Cell Signaling Technology, 12885), GluA1 (SYSY, 182011).

Paraffin sections of human brain tissues were provided by: Human Brain Bank, Chinese Academy of Medical Sciences & Peking Union Medical College, Beijing, China. Immunofluorescence staining was used to study the distribution of DIR and Aβ. In a single tissue section, primary antibodies raised in different species (mouse, rabbit or sheep) were used. The secondary antibodies (anti-mouse, anti-rabbit or anti-sheep) were coupled to different fluorochromes (Alexa Fluor 488, Cy3 or Cy5). After staining, cover the fluorescently labeled sections with VECTASHIELD mounting medium (Vector Labs). The primary antibodies and dye used were listed here: Aβ (Cell Signaling Technology, 8243S), Thioflavin S (ChemCruz, sc-391005), Gelsolin (Abcam, ab109014).

**RNA-seq Data and processing**

The Alzheimer’s disease human brain transcriptomes were obtained from the National Center for Biotechnology Information (NCBI). The accession number is GSE153873 and GSE159699. The tissue type all is postmortal lateral temporal lobe. The average sequencing depth of the subjects was 25 to 30 million reads. The data were aligned to the human reference genome (version 110 of GRCm38) using STAR.

**Quantification of intron retention using IRFinder**

For using IRFinder, we first built a reference using ‘IRFinder BuildRef’ command based on the Release 110 of Ensembl human genome GRCh38 gene annotations, including RNA.SpikeIn.ERCC.fasta.gz and Human_hg38_nonPolyA_ROI.bed files obtained from IRFinder repository (https://github.com/RitchieLabIGH/IRFinder). After downloading the transcriptome FASTQ files as described in the previous subsection, we quantified the intron retention via ‘IRFinder FastQ’ command. The IR ratio was calculated as following formula:


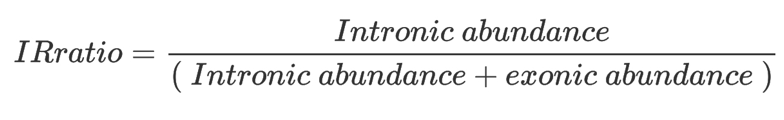
.

**NPCs and neuronal differentiation**

Human embryonic stem (ES) cells were used to be differentiated into neural progenitor cells (NPCs) and then differentiated into neurons. The NPCs were maintained in Dulbecco’s modified eagle’s medium: F12 (DMEM: F12, Thermo Fisher) containing 1×B27 (Sigma-Aldrich), 1×N2 (Gibco), and basic fibroblast growth factor (20 ng/mL, bFGF, Sigma-Aldrich) at 37°C with 5% CO_2_. The NPCs were differentiated into neurons in the absence of bFGF for at least 3 weeks and treated with 10 μM ROCK inhibitor (Y-27632; Calbiochem) during the first 24 h.

**Plasmid construction and expression**

Primers, inducing the extra homology arms paired with the pCMV-flag vector constructed based on the pCMV-myc vector, were designed to clone the full length of DIR CDS sequences.

Forward: 5’-CCATGGAGGCCCGAATTCGGATGGTTGCAACTGGC-3’;

Reverse: 5’-ACTCATCAATGTATCTTATCTTATGGAGAGAAGATGTTAGAAAA-3’.

Primers, inducing the extra homology arms paired with the pcDNA3.1-myc-his vector, were designed to clone the full length of human Gelsolin CDS sequences.

Forward: 5’-GTGGAATTCGCCACCATGGCTCCGCACCGCCC-3’;

Reverse: 5’-CCCTCTAGACTCGAGGGCAGCCAGCTCAGCCATGG-3’.

Primers, inducing the extra homology arms paired with the pcDNA3.1-myc-his vector, were designed to clone the full length of human GluA1 CDS sequences.

Forward: 5’-TGGCTAGTTAAGCTTGCCACCATGCAGCACATTTTTGCCTTCTT-3’;

Reverse: 5’-TGCTGGATATCTGCAGAATTCCAATCCCGTGGCTCCCAAGGGCAT-3’.

Then, the sequence and linearized pCMV-flag, pCMV-myc and pcDNA3.1-myc-his vector were recombined using Hieff Clone^®^ Plus One Step Cloning Kit (Yeasen Biotechnology (Shanghai) Co.). Next, the expression vector (pCMV-flag-DIR, pcDNA3.1-GluA1-myc-his and pcDNA3.1-Gelsolin-myc-his) and the control (pCMV-flag and pcDNA3.1-myc-his) were transfected to HEK293 cells with the help of PEI40000 reagent (Yeasen Biotechnology (Shanghai) Co.). The cells were further cultured for 48 h, and the DMEM mediums (Invitrogen) containing 10% FBS (Yeasen Biotechnology (Shanghai) Co.) were collected and centrifuged with 1000 rpm velocity for 10 min at 4°C. Then, the supernatants were transferred for Western blot assay. On the other hand, the transfected cells were lysed with the HEPES lysis buffer (30 mM HEPES, 150 mM NaCl, 10 mM NaF, 1% Triton X-100, and 0.01% SDS) and centrifuged with 12000 rpm velocity for 10 min at 4°C, and the supernatants were transferred for Western blotting.

**Protein purification**

Gelsolin purification: pcDNA3.1-gelsolin-myc-his plasmids were transfected to HEK293 cells. After 48 h later, the cell culture medium was collected and centrifuged for 10 min with the speed of 12000 rpm. The supernatant was transferred and then incubated with Ni-NTA agarose resins (Yeasen, 20503ES60). The resins were washed with Buffer A (50 mM NaH_2_PO_4_, 300 mM NaCl, and 10 mM imidazole, PH = 8.0) before binding with supernatant. Buffer B (50 mM NaH_2_PO_4_, 300 mM NaCl, and 20 mM imidazole, PH = 8.0) was used to wash the resins after incubation. The attached proteins were eluted from resins using Buffer C (50 mM NaH_2_PO_4_, 300 mM NaCl, 250 mM imidazole, PH = 8.0). The eluted solution was concentrated using Millipore Amicon® Ultra-15 and was replaced by PBS. The purified gelsolin was stored in-80°C refrigerator.

GluA1 purification: the GluA1 CDS was clone to the expression vector pGEX-4T1-GST using the following primers.

Forward: 5’-CGGCCGCATCGTGACGCCAATTTCCCCAACAATATCCAGA-3’;

Reverse: 5’-GCAGATCGTCAGTCACAATCCCGTGGCTCCCAAGG-3’.

The pGEX-4T1-GST-GluA1 recombinant plasmid was transformed into *E. coli* BL21. Then a single clonal *E. coli* was cultured in Luria-Bertani medium (LB) medium until OD_600_ = 0.6~0.8. Next, 0.5 mM isopropyl-β-D-thiogalactopyranoside (IPTG, Sigma) was added to induce GST-GluA1 expression under 18°C for 16 h. The medium was centrifuged with 6000 rpm for 15 min at 4°C. The pellet was lysed with Lysis Buffer (20 mM Tris, 5 mM DTT, and 8 M urea, pH = 8.0) followed by sonification. After centrifugation, the supernatant was diluted to 1 M urea using Equilibrium Buffer (20 mM Tris-HCl, and 0.15 M NaCl, pH = 8.0) and then loaded onto a glutathione-agarose column previously equilibrated on Equilibrium Buffer. Wash Buffer (20 mM Tris, 1 mM EDTA, 1 M NaCl, and 0.5% Triton X-100, pH = 8.0) was supplied to wash away uncombined proteins and the GST-GluA1 was eluted using Elution Buffer (20 mM Tris-HCl, 50 mM GSH, and 0.15 M NaCl, pH = 8.0). The eluted solution was concentrated using Millipore Amicon® Ultra-15 and was replaced by PBS. The purified GST-GluA1 was stored in -80°C refrigerator.

DIR purification: the DIR CDS was clone to the expression vector SUMO-Strep using the following primers.

Forward: 5’-GAAAATTTGTACTTCCAGGGCATGGTTGCAACGGGTTCCCTG-3’;

Reverse: 5’-GGATGGCTCCACGGGCTGAAAATGTTGCTGAAC-3’.

The SUMO-DIR-Strep was expressed and purified from *E. coli* BL21. The transformed *E. coli* BL21 cells were cultured in LB at 37℃ until the OD_600_ = 0.6~0.8, and then were added 0.5 mM IPTG for the induction of protein expression at 18°C for 16-18 h. Cells were harvested and resuspended in lysis buffer (50 mM Na_2_HPO_4_, 300 mM NaCl, 1% SDS, and 1 mM PMSF, pH = 8.0) and lysed by sonication. The lysate was cleared by centrifugation at 30,000 g for 30 min at 4°C. The supernatant was loaded on a Streptactin Beads 4FF column (Smart-Lifesciences) pre-equilibrated with lysis buffer. The column was washed with 200 ml wash buffer (50 mM Na_2_HPO_4_ pH 8.0, and 300 mM NaCl) and eluted with elution buffer (50 mM Na_2_HPO_4_ pH 8.0, 300 mM NaCl, and 3 mM D-desthiobiotin). Then proteins were concentrated and equilibrated with 1×PBS. Finally, the protein concentration was estimated by UV at 280 nm, frozen in liquid nitrogen and stored at -80°C.

**Mass spectrometry (MS)**

For MS analysis, the in-gel digestion was performed by using the following protocol. The strip was performed in 1% 1, 4-dithiotreitol (DTT) in the SDS equilibration buffer (50 mM Tris-Cl (pH 8.8), 6 M urea, 30% glycerol, 2% SDS and bromophenol blue) for 15 min. This step was followed by alkylation of the free sulhydryl groups by 2.5% iodoacetamide in the SDS equilibration buffer for another 15 min in the dark at room temperature. Then the strip was cut into 18 gel sections (each section about 1.0 cm in length). Each gel section was washed three times alternately with acetonitrile and 100 mM ammonium bicarbonate. During the last wash the gel slices were incubated in 100 mM ammonium bicarbonate for 15 min at 4°C. The gel slices were dried by vacuum centrifugation and allowed to swell in a 50 μl trypsin solution containing trypsin (20 μg/ml) and ammonium bicarbonate (50 mM) for 45 min at 4°C. After adding another 50 μl of trypsin solution, the gel slices were kept for 20 h at 37°C. The supernatant was transferred to another vial, and the gel slices were extracted for 15 min three times by 0.1% formic acid in 60% acetonitrile. The recovered peptide solutions were dried by vacuum centrifugation and desalted and cleaned using a Ziptip (Millipore, Corp., Bedford, MA).

The peptide mixtures from each section of the strip were separated by Reverse phase HPLC (RP-HPLC) followed by tandem mass analysis. RP-HPLC was performed on a surveyor LC system (Thermo Finnigan, San Jose, CA). The C18 column (RP, 180 μm × 150 mm) was obtained from Column Technology Inc. (Fremeont, CA). The pump flow was split 1:120 to achieve a column flow rate of 1.5 μl/min. Mobile phase A was 0.1% formic acid in water, and mobile phase B was 0.1% formic acid in acetonitrile. The tryptic peptide mixtures were eluted using a gradient of 2-98% B over 180 min.

The MS was performed on a LTQ linear ion trap mass spectrometer (Thermo Finnigan, San Jose, CA) equipped with an electrospray interface and operated in positive ion mode. The capillary temperature was set to 170°C and the spray voltage was at 3.4 kV. Normalized collision energy was at 35%. Automatic gain control was used to obtain maximal signal of each scan. The mass spectrometer was set so that one full MS scan was followed by ten MS/MS scans on the 10 most intense ions. Dynamic exclusion was set at repeat count 2, repeat duration 30 s, and exclusion duration 90 s.

The acquired MS/MS spectra was searched against the IPI human database using BioWorks 3.0 software (Thermo Finnigan) on an 8 node Dell PowerEdge 2650 cluster. An accepted SEQUEST result had a ΔCn score of at least 0.1 (regardless of charge state), a value known for high confidence in a SEQUEST search. All output results were combined by using a homemade software named Build Summary to delete the redundant data. To make sure that the MS/MS spectrum was of good quality with fragment ions clearly above baseline noise, we referred to the parameters reported in previous studies and applied stricter criteria for the peptide identification. Peptides were validated after meeting the following criteria. The SEQUEST cross-correlation score must be ≥ 1.9 for a + 1 tryptic peptide, ≥ 2.2 for a + 2 tryptic peptide and ≥ 3.75 for a + 3 tryptic peptide. In addition, ΔCn cutoff values were ≥ 0.1 and the SP rank of the peptides ≤ 4.

**ThT fluorescence assay**

ThT binding assays were modified from the previously reported article. Briefly, Monomeric Aβ42 (1 mM) was dissolved with DMSO and diluted to a final concentration of 10 μM with ThT fluorescence assay buffer (50 mM sodium phosphate buffer (pH = 7.4), 50 mM NaCl, 1 μM ThT and 0.01% sodium azide). Samples were added to a 96-well black plate and incubated at 37 °C without shaking. Real-time ThT fluorescence measurements were taken using a Tecan Spark microplate reader (Tecan). The fluorescence values were measured every 10 - 20 min for 3 - 4 h at excitation and emission wavelengths of 440 nm and 480 nm, respectively.

**Direct DIR antibody ELISA Assay**

Mice were randomly assigned to two experimental groups: IgG and DIR antibody (0.5mg/mouse, iv). Animals in the intravenous injection group were anesthetized 30 minutes post-injection, followed by intracardially perfused with ice-cold PBS and rapid extraction of hippocampal and cortical tissues. Tissues were immediately placed on ice. Harvested tissues were homogenized in ice-cold phosphate-buffered saline (PBS) containing protease inhibitors at a ratio of 10 µL per mg of tissue. The homogenates were centrifuged at 13,000g for 10 minutes at 4°C to pellet debris, and the supernatants were collected. Protein concentrations were determined using a BCA protein assay kit, ensuring accurate quantification for ELISA.

Microplate wells were coated with DIR-Intron peptide and biotinylated IgG or DIR antibody with coating buffer. Plates were incubated at 37 °C for 1 hour. Following incubation, the plates were washed to remove unbound material and blocked with blocking buffer for 1 hour at room temperature. After blocking, wells were incubated with streptavidin-horseradish peroxidase (SA-HRP) conjugate, diluted according to the manufacturer's instructions, for 1 hour at 37 °C. The plates were then washed again to remove unbound conjugate. Tetramethylbenzidine (TMB) substrate solution was added to each well, and the plates were incubated in the dark until a color change was observed. The reaction was stopped with an acidic stop solution, and the absorbance at 450 nm was measured using a microplate reader. A standard curve was constructed using the optical density (OD) values of the biotinylated peptide standards. The concentration of biotinylated peptides in the tissue lysates was interpolated from the standard curve.

**Brain slice preparation**

Adult mice were anaesthetized and decapitated, then the brains were quickly removed and placed in ice-cold artificial cerebrospinal fluid (ACSF) containing the following compounds (in mM): 117 NaCl, 3.6 KCl, 1.2 NaH_2_PO_4_⋅2H_2_O, 2.5 CaCl_2_⋅2H_2_O, 1.2 MgCl_2_⋅6H_2_O, 25 NaHCO_3_, and 11 glucose. The ice-cold ACSF had a PH of 7.4 when bubbled with 95% O_2_ and 5% CO_2_. The brains were glued onto the stage of a Leica VT1200S vibratome and 350 μm-thick transverse hippocampal slices were cut. The slices were incubated for at least 1 h in oxygenated (95% O_2_ and 5% CO_2_) ACSF at room temperature before recording.

**Electrophysiology**

For spontaneous excitatory postsynaptic current (sEPSC), the slices were transferred to a submerged chamber that was mounted on the stage of an upright microscope (Olympus, BX51WIF), and perfused with oxygenated (95% O_2_ and 5% CO_2_) ACSF. The patch pipettes (2.5 – 3.5 MΩ) were fabricated from borosilicate glass tubes (WPI, 1B150F-3) using a horizontal puller. The pipettes solutions contained (mM): 135 K-gluconate, 0.5 CaCl_2_, 2 MgCl_2_, 5 KCl, 5 EGTA, 5 HEPES, and 5 D-glucose (PH adjusted to 7.3 with KOH). Spontaneously occurring excitatory postsynaptic currents (sEPSCs) of hippocampal CA1 region recordings were performed using an amplifier (Multiclamp 700B, Molecular Devices) under the voltage clamp, with cells clamped at -70 mV. Signals were filtered at 2 kHz. All currents were sampled using a Digidata 1,550B interface. Traces were recorded in pClamp10.5 software (Molecular Devices).

For the long-term potentials, hippocampal slices were placed on submersion recording chambers perfused with oxygenated (95% O_2_ and 5% CO_2_) ACSF (30 ± 0.5℃, 2 ml/min). fEPSPs responses were evoked by stimulating the Schaffer collateral with 0.2 ms pulses delivered through customized double barrel glass electrodes (Sutter Instrument Co.) and recording from stratum radiatum of area CA1 using glass pipettes filled with ACSF. The stimulation intensity was systematically increased to determine the maximal fEPSP slope and then adjusted to half maximal fEPSPs slope. fEPSPs were recorded, filtered at 0 – 1000 Hz, digitized and stored at 5K sampling rate using customized program in Spike hound. fEPSPs slope were analysed using customized Matlab code. Initial slopes of fEPSPs were expressed as percentages of baseline averages. After 30 min stable baseline recorded, LTP was induced by 4 trains of theta-burst stimulation (TBS) at 0.1 Hz. Each train consists of 10 bursts (4 pulses at 100 Hz per burst) repeated at 5 Hz.

**Transmission Electron Microscope (TEM)**

For TEM, two hundred microliters of 5 μM monomeric Aβ42 alone, 5 μM monomeric Aβ42 and 1 μM DIR-Intron peptide added with or without 0.1 μM gelsolin were incubated overnight in RIPA lysis buffer at 4°C. Then, the samples were centrifugated (15,000 g for 5 min at 4°C) to collect the pellets and resuspended with twenty microliters of RIPA lysis buffer. The TEM samples were bound to carbon-coated grids and stained with 2% uranyl formate. Finally, images were collected using TEM (Thermo Fisher Scientific) operated at 120 kV.

**Proximity ligation assay (PLA)**

To test interaction of DIR, gelsolin and Aβ in situ, paraffin section of human brain tissues was provided by Human Brain Bank, Chinese Academy of Medical Sciences & Peking Union Medical College, Beijing, China. Slides were blocked and then incubated in a humidity chamber for 60 min at 37°C. Then the antibodies recognized DIR, gelsolin and Aβ were supplied to stain the slides overnight at 4°C. On the next day the slides were incubated with PLUS and MINUS PLA probes (1:5; Sigma; DUO92001 and DUO92005) for 1 h at 37°C followed by ligation with 1X Ligation Buffer (1:40; Sigma; DUO92008) and the additional incubation for 30 min at 37°C. Moreover, the slides were incubated with Amplification-Polymerase solution (1:80; Sigma; DUO92008) for 100 min at 37°C. Finally, mount the slides with a cover slip using a minimal volume of Duolink In Situ Mounting Medium with DAPI (Sigma; DUO82040). Leica SP8 microscope was used for imaging.

**Hypoxia treatment**

Neural stem cells and differentiated neurons were placed in a chamber system (Stemcell^TM^ technologies) with the hypoxic condition of 1% O_2_ and 21% O_2_ as control condition for 24 h. Then, the cells were lysed with the Lysis Buffer (100 mM Tris, 150 mM NaCl, 8 M urea and protease inhibitors, PH = 7.5) followed by sonification, and the lysates were centrifuged with 12000 rpm velocity for 10 min at 4°C, and the supernatants were measured with Western blotting.

Mice were accommodated in laboratory mouse cage continues supplied with hypoxic condition (8% O_2_) through air inlet hole for 8 h. Oxygen analyzer (Shanghai Precision Instrument Co., Ltd) were detected in air outlet hole ensuring the hypoxic condition of the cage.

**Animal model of cerebral ischemia**

The ischemia model based on middle cerebral artery occlusion (MACO) surgery, was modified according to the published paper. Briefly, mice were anesthetized with isoflurane and then a skin lesion in the middle of the neck was cut. After gently separating the muscle, the common carotid artery (CCA) and its branches were exposed under a stereo dissecting microscope. Then the CCA was gently ligated by silk sutures to slower the blood flux while the distal external carotid artery (ECA) was tightly ligated to block the flux. Next, a small incision of the ECA between the branch point and ligation was generated and the tip rounded suture (Beijing Cinotech Co.) was inserted along the ECA into the internal carotid artery (ICA) until to occlude the origin of middle cerebral artery (MCA). Furthermore, tightly ligation between incision and branch point of ECA was performed while the suture was slowly withdrawn from CCA. Finally, the surgical region was closed.

***In vivo* injection**

The DIR-Intron was released to the bilateral hippocampus of mouse brain by osmotic pumps system (ALZET®, 1002). Briefly, pumps were implanted subcutaneously to direct deliver the DIR-Intron peptide or the scrambled peptide (dissolved in PBS) to the injection site via the brain infusion kit (ALZET®) for two weeks. mDIR siRNAs (SS- GCGUUGUUCCAGUCUGCUATT; AS-UAGCAGACUGGAACAACGCTT) were purchased from Guangzhou RiboBio Co., Ltd. In total, 15 nmol unit siRNAs were injected to bilateral hippocampi of WT or APPPS1 mice for 3 times with 3 days intervals between each injection. Injection sites were the CA1 regions of bilateral hippocampi (from bregma, siRNAs were injected ± 2.0 mm lateral, -2.25 mm caudal, -1.85 mm ventral).

***In vivo* two-photon imaging**

Three to 5-month-old mice were anesthetized with 1.5% isoflurane and fixed to a stereotaxic frame with custom-built stage-mounted ear bars. Then a cranial window was implanted on the head of mouse. A 1.5 cm incision was made between the ears, and the scalp was reflected to expose the skull followed by making a circular craniotomy (5 – 6 mm diameter) using a high-speed drill and a dissecting microscope for gross visualization. Next, a glass-made coverslip was attached to the skull. Then, anti-DIR antibodies were conjugated with FITC (Abcam, ab188285) under the product instructions. Finally, a two-photon microscope turned to 920-nm and 1045-nm wavelength lasers were used to acquire images after intravenous injection of Dextran-Texas Red (Thermo Fisher Scientific, D1830) and DIR antibodies.

**Behavior phenotyping**

*Y-maze:* The animals were placed in one arm end of three identical arms (40 × 40 × 40 cm with the 120° angle between arms) and given free access to the arms for a 10-min session. The entry of mice into each arm was video-recorded, and the sequence and number of arms entered were analyzed.

*Novel object recognition test:* Adult mice were placed in and familiar with an open box (40 × 40 × 40 cm). 12~24 h later, two identical objects were set to the box and mice were allowed to explore for 10 min. To test for object recognition after training, one of the objects was replaced with a novel object, and the animals were entered in the apparatus again for 10 min. The time spent on exploring each of the objects was measured. All movement were recorded by a camera.

*Morris water maze:* Morris water maze was performed as previously described, but with minor modifications. The experimental procedures consisted of 2 phases: training phase and test trials. The training phase consisted of 4 trials daily with different entries (30-min interval) for 5~6 days. A hidden platform was set 0.5 cm under the water. Mice were allowed to swim freely to find the platform for 1 min. If not completed within the specified period, the correct routes would be offered by operators. Mice were allowed to stay on the platform for 30 s before being picked up. The test trials were performed after training, mice were placed in water and the platform was removed. The escape latency and the time spent in each quadrant were recorded by a camera and were analyzed with Etho Vision XT 14 software.

*Open-field test:* Mice were placed in an open field (45 × 45 cm) and recorded the exploratory locomotor activity in a 30 min period by a Digiscan apparatus (Accuscan Electronics). The moving distance and velocity were analyzed.

**Statistical Analysis**

The data are presented as mean ± SEM. Sample number (n) values are indicated in figures, figure legends or results section. Two groups were compared by a two-tailed, unpaired Student’s t test. Comparisons between two groups with multiple time were performed by a two-way ANOVA with Bonferroni’s post hoc test.

Statistical analysis was performed using PRISM (GraphPad Software). Differences were considered significant at p < 0.05 (*p < 0.05, **p < 0.01, ***p < 0.001).

**Supplementary figure and legend**

**
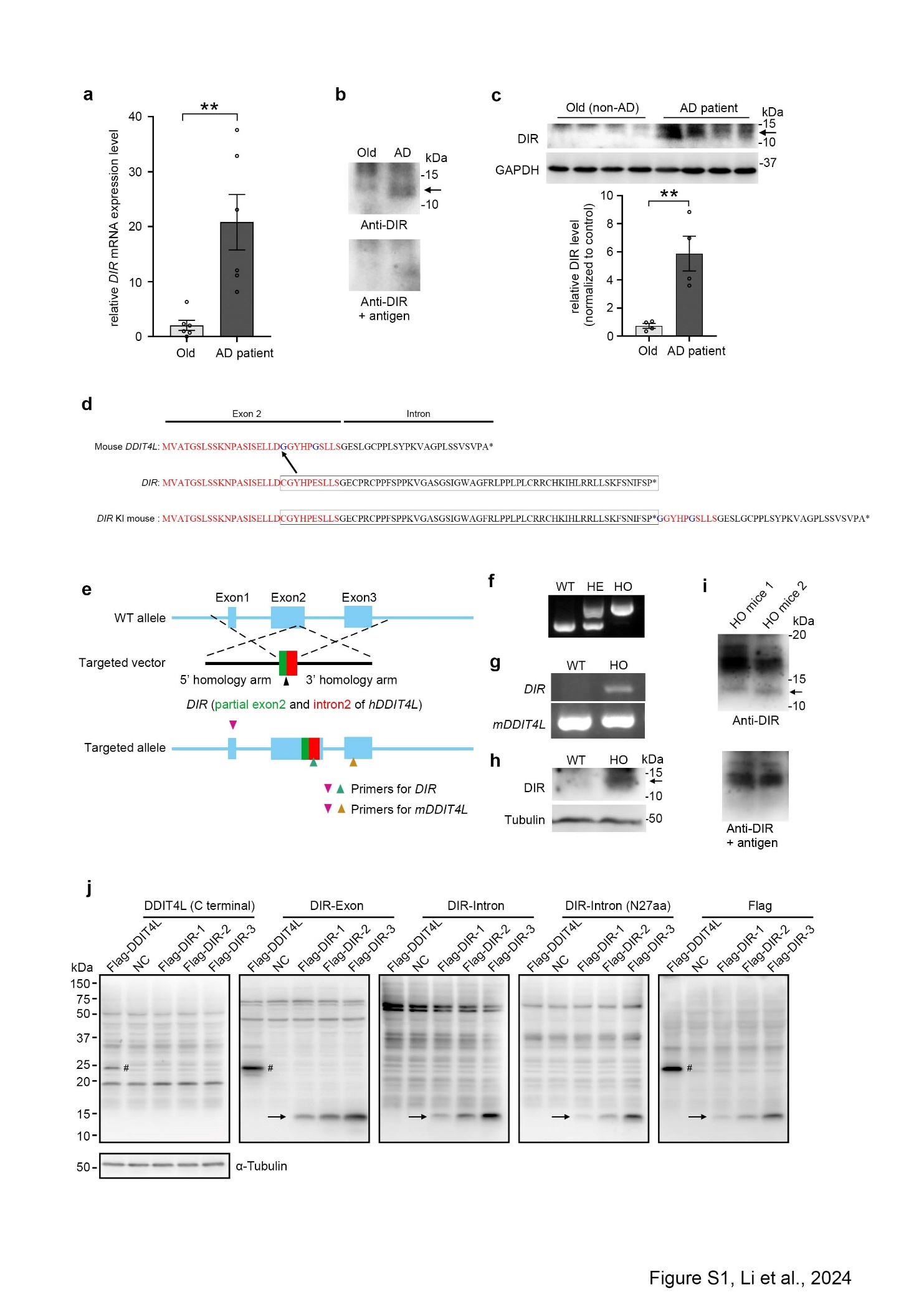
**

**Fig. S1, DIR expression in in AD patients, construction of DIR-KI mouse, and verification of DIR-KI mice**

1. Increase in the expression of DIR mRNA (normalized to DDIT4L) in the AD patients’ hippocampi as compared with that of non-AD old people. **, p < 0.01.
2. Top: the DIR antibody detected an immunoreactive band (~12 kDa, arrowheads) of DIR protein in AD patients.

Bottom: the pre-absorbed DIR antibody failed to detect the immunoreactive band (~12 kDa, arrowheads) of DIR protein in AD patients.

1. Immunoblotting showed the relative expression of DIR protein in non-AD old people and AD patients. Statistical result showed the increased expression of DIR in AD patients as compared with that of non-AD old people. **, p < 0.01.
2. Comparison between human and mouse DDIT4L sequences.
3. The *DIR* knock-in (KI) mouse was constructed via CRISPR/Cas9 assays. To ensure the correct and intact expression of human *DIR*, the human *DIR* sequence encoding the last 64 AAs (the green block showing the 21-30 AAs of human DDIT4L, the red one for the 31-84 AAs of the DDIT4L intron translated region) was inserted behind the mouse *DDIT4L* genome that translated 1-20 AAs.
4. Genotyping and sequencing of newborn mice confirmed the accurate sequence insertion of *DIR* (WT: wild-type, HE: heterozygote, HO: homozygote).
5. The designed primers identified the human DIR mRNA in the HO mice.
6. Immunoblotting showed that DIR protein (~12 kDa, arrowheads) was detected in the hippocampus of HO mice by the DIR antibody.
7. Immunoblotting showed an immunoreactive bands (~12 kDa, arrowheads) detected with the antibody against DIR, but not by the pre-absorbed antibody in the hippocampus tissue of DIR-KI mice (HO).
8. In HEK293 cells, transfected Flag-DDIT4L (2 μg), Flag-DIR-1 (1 μg), Flag-DIR-2 (2 μg), Flag-DIR-3 (3 μg), then the antibodies against C-terminal of DDIT4L, DIR-Exon, DIR-Intron, DIR-Intron (N27aa, 27aa of Intron N-terminal) and Flag. The arrowhead shown special band for DIR, the # signal shown special band for DDIT4L.

Data shown are mean ± S.E.M. Two-tailed unpaired *t*-test (a, c).


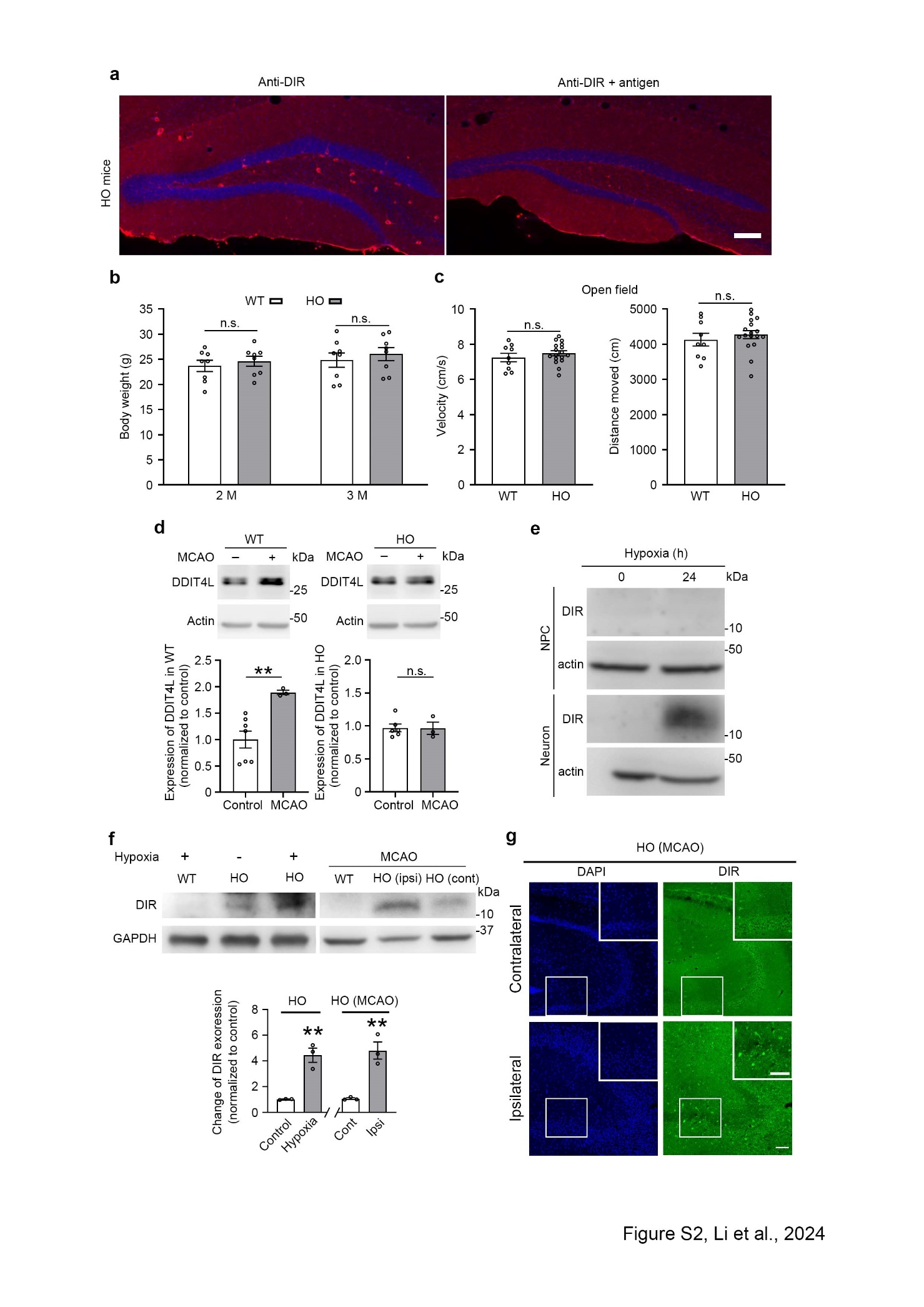


**Fig. S2, Increased DIR expression in hippocampi of WT and DIR-KI mice under hypoxia**

1. The antigen absorption of DIR antibody abolished the DIR-immunoreactive signals in the hippocampus of homozygous DIR-KI mice. Scale bar = 50 μm.
2. The body weight of the homozygous DIR-KI mice was not different from that of WT mice.
3. The open field test showed no difference in the moving distance and velocity between WT mice and the homozygous DIR-KI mice.
4. The mDDIT4L expression was increased in the hippocampus of MCAO WT mice. **, p < 0.01. n.s., no significance.
5. The neurons derived from human stem cells, not the neural precursor cells (NPC), enhanced the expression of DIR under hypoxia (1% O_2_, 24 h).
6. The immunoblotting showed the DIR expression in hippocampus was increased in the HO mice exposed to the hypoxia condition (8% O_2_, 8 h). The DIR expression was apparently increased in the ipsilateral (Ipsi) versus the contralateral (Cont) hippocampus in MCAO hypoxic-ischemic brain injury model of HO mice. **, p < 0.01.
7. Immunostaining showed the increased expression of DIR in the ipsilateral hippocampus in MCAO hypoxic-ischemic brain injury model of homozygous DIR-KI mice (n = 4). Scale bar = 100 μm.

Data shown are mean ± S.E.M. Two-tailed unpaired *t*-test.


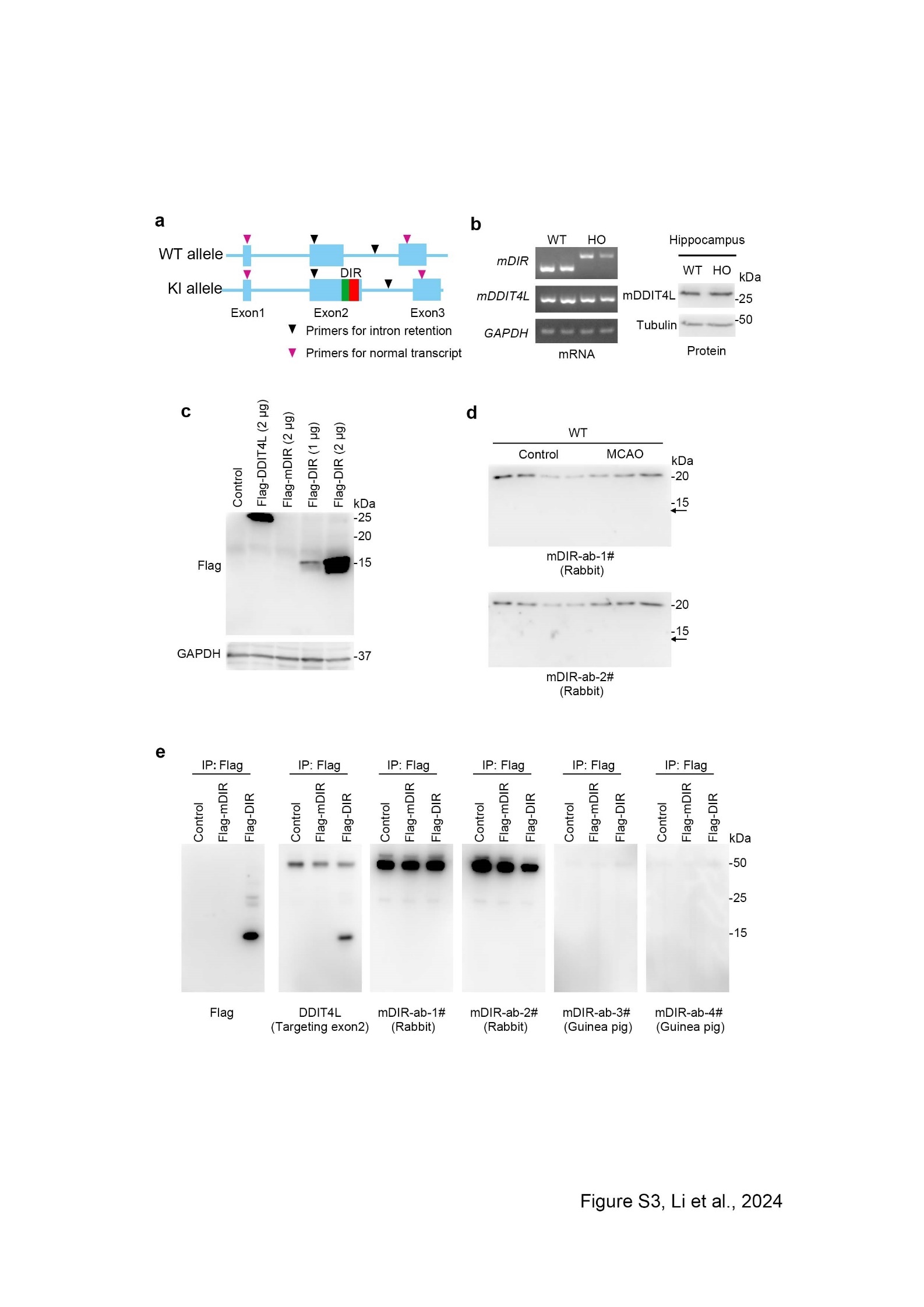


**Fig. S3, Detection of DIR and mDIR**

1. Schematic diagram about primers for detecting the intron retention and normal transcript of DDIT4L.
2. Specific primers confirmed the intrinsic expression of intron retention of DDIT4L (mDIR) in WT and HO mice. The mDDIT4L expression in the hippocampus of HO mice was not changed as compared with that of WT mice.
3. Immunoblotting showed that Flag antibody could not detect Flag-mDIR in the lysate of HEK293 cells transfecting Flag-mDIR plasmid.
4. The mDIR was not detected in the hippocampus of normal control and MCAO model of WT mice by two different antibodies.
5. The immunoprecipitation showed that Flag-DIR was detected by Flag antibody and DDIT4L antibody in the lysate of transfected HEK293T cells, indicating that *DIR* mRNA was translated to the protein. However, the Flag-mDIR could not be detected by Flag, DDIT4L or mDIR antibodies in the lysate of transfected HEK293T cells, suggesting the *mDIR* mRNA was not translated to the protein.


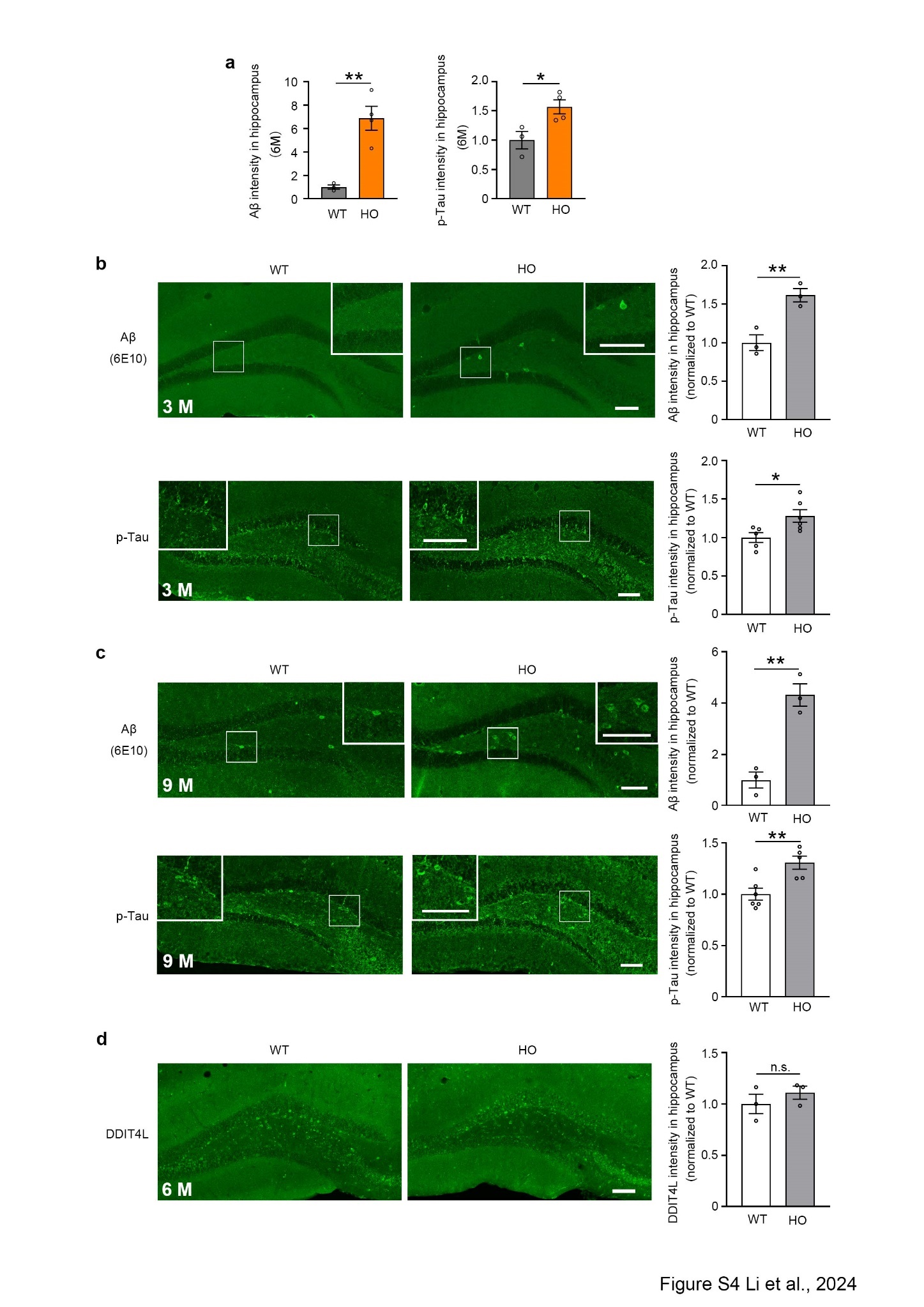


**Fig. S4, Aβ and p-Tau expression in 3- and 9-month-old DIR-KI mice, and the DDIT4L expression in 3-month-old DIR-KI mice**

1. Statistical results showed that the expression of Aβ and p-Tau was upregulated in the hippocampal dentate gyrus in the 6-month-old homozygous DIR-KI mice. *, p < 0.05. **, p < 0.01.
2. The representative images and statistical results showed that the expression of Aβ and p-Tau was upregulated in the hippocampal dentate gyrus in the 3-month-old homozygous DIR-KI mice. Scale bars = 100 μm. *, p < 0.05. **, p < 0.01.
3. The representative images and statistical result showed that the expression of Aβ and p-Tau was increased in the hippocampal dentate gyrus in the 9-month-old homozygous DIR-KI mice. Scale bars = 100 μm. **, p < 0.01.
4. The representative images and statistical result showed that the expression of DDIT4L was not changed in the hippocampal dentate gyrus in the 6-month-old WT and homozygous DIR-KI mice. Scale bars = 100 μm. n.s., no significance.

Data shown are mean ± S.E.M. Two-tailed unpaired *t*-test.

**
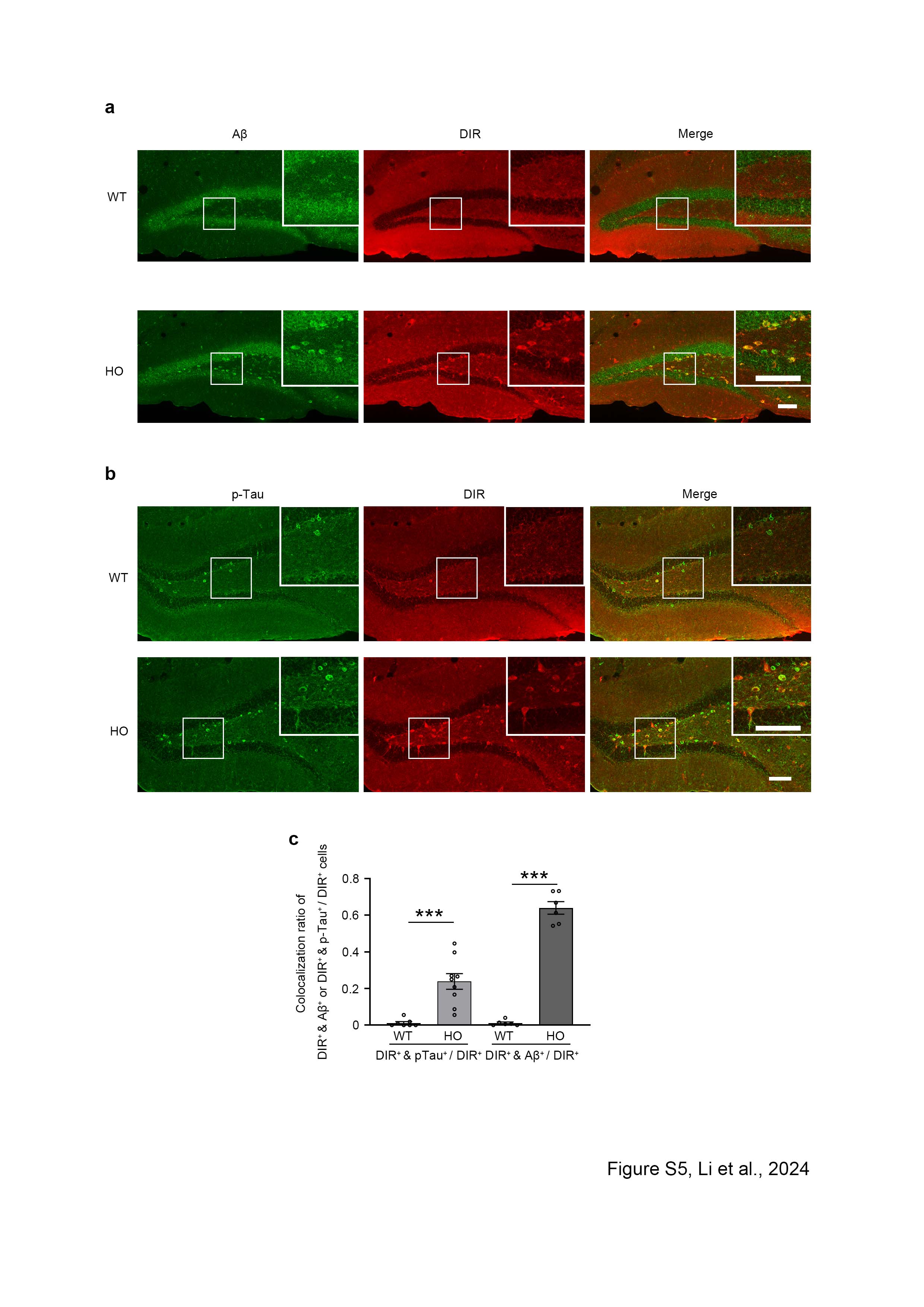
**

**Fig. S5, DIR colocalization with Aβ and p-Tau**

1. The representative images showing that the immunoreactive signals of DIR and Aβ were colocalized in the hippocampal dentate gyrus of 6-month-old DIR-KI mice. Scale bars = 100 μm.
2. The representative images showing that the immunoreactive signals of DIR and p-Tau were colocalized in the hippocampus of 6-month-old DIR-KI mice. Scale bars = 100 μm.
3. Quantificational analysis showed that DIR could be colocalized with Aβ and p-Tau in the hippocampus of 6-month-old DIR-KI mice. ***, p < 0.001.

Data shown are mean ± S.E.M. Two-tailed unpaired *t*-test.


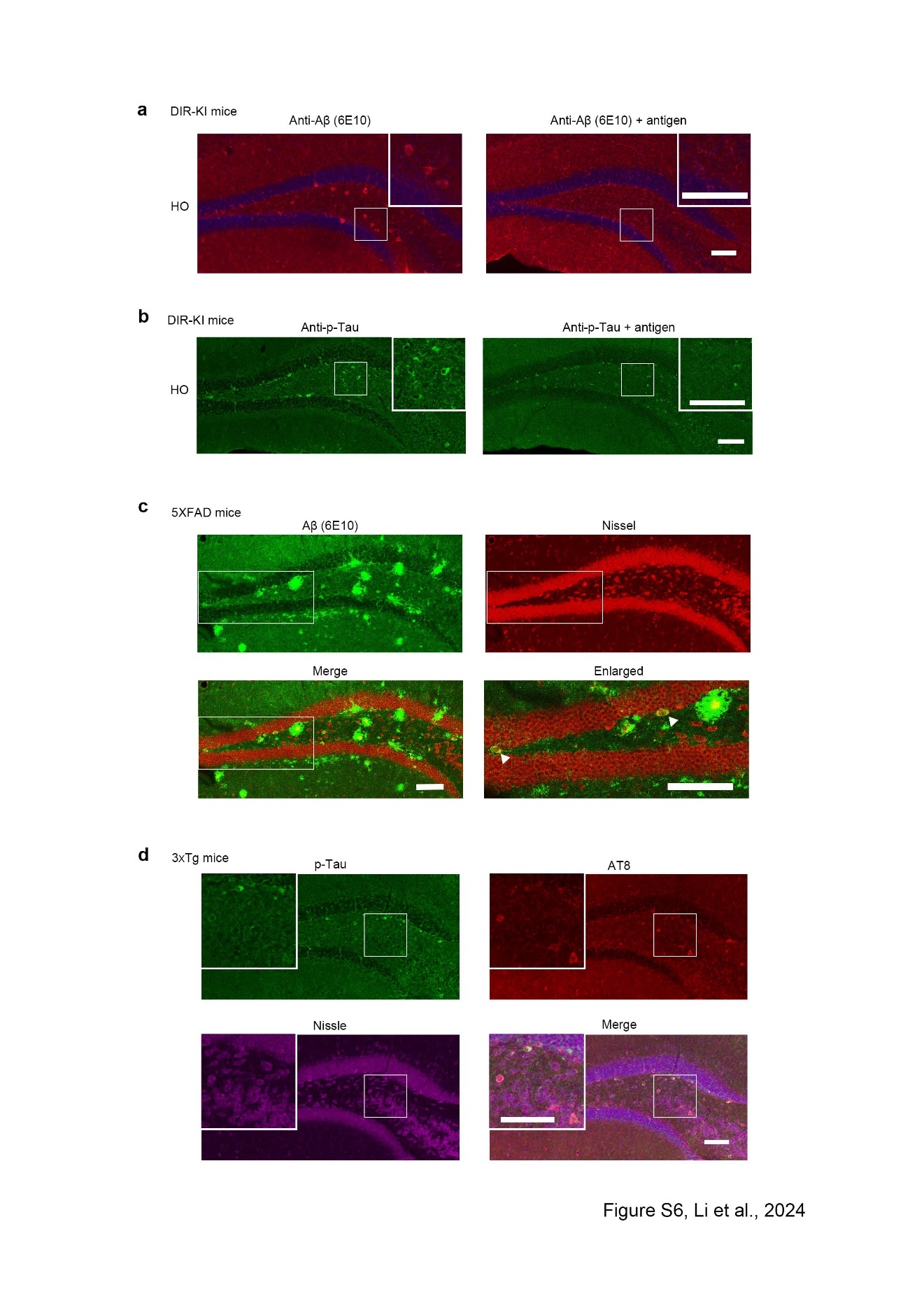


**Fig. S6, Verification of Aβ and p-Tau antibodies**

1. The antigen absorption of Aβ antibody (6E10) abolished the immunostaining signals in the hippocampus of 6-month-old DIR-KI mice. Scale bars = 100 μm.
2. The antigen absorption of p-Tau antibody abolished the immunostaining signals in the hippocampus of 6-month-old DIR-KI mice. Scale bars = 100 μm.
3. The Aβ antibody (6E10) detected Aβ in the hippocampal neurons of 6-month-old 5XFAD mice. Scale bars = 100 μm.
4. The p-Tau antibody detected AT8-positive neurons in the hippocampus of 6-month-old 3XTg mice. Scale bars = 100 μm.


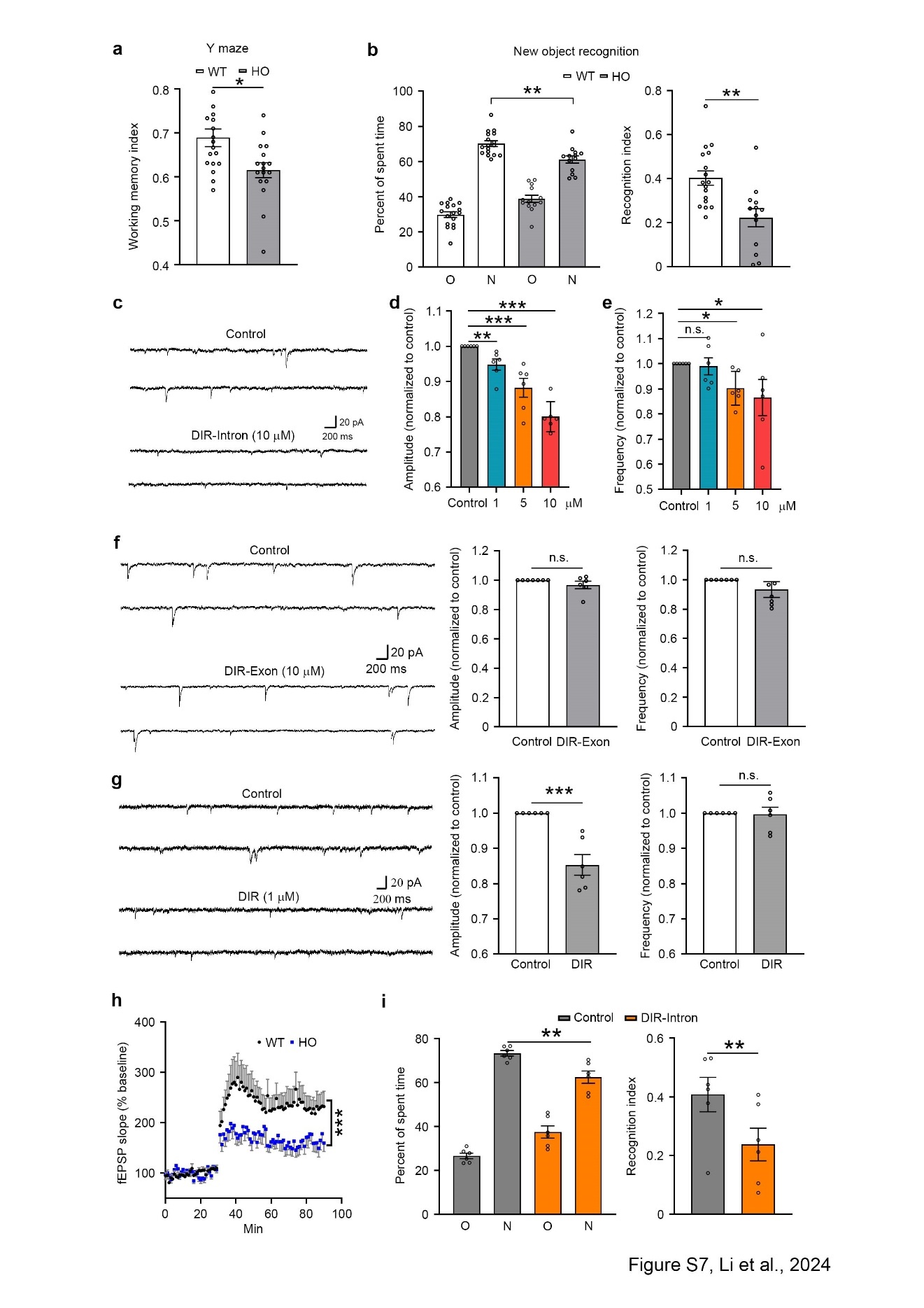


**Fig. S7, Working memory and novel object recognition of DIR-KI mice, and DIR- and DIR-Intron-induced enhancement of sEPSC and LTP in hippocampi**

1. The Y maze alternative test showed that the working memory was impaired in the homozygous DIR-KI mice (HO), as compared with that of wild-type mice (WT). *, p < 0.05.
2. The decreased spent time and recognition index showed that the capacity of novel object recognition was impaired in the homozygous DIR-KI mice compared with that of WT mice. O, old object; N, new object. **, p < 0.01.
3. The representative traces showed the inhibitory effects after DIR (10 μM) incubation on the amplitude and frequency of spontaneous excitatory postsynaptic current (sEPSC) of hippocampal neurons in the brain slices prepared from WT mice.
4. The statistical result showed that the amplitude of sEPSC in hippocampal neurons was suppressed by the DIR-Intron in a dose-dependent manner. **, p < 0.01. ***, p < 0.001.
5. The statistical result showed that the frequency of sEPSC in hippocampal neurons was dose-dependently reduced by the incubation with the DIR-Intron. *, p < 0.05. n.s., no significance.
6. The representative traces and statistical results showed that the amplitude and frequency of sEPSC of hippocampal neurons were not altered by applying DIR-Exon (10 μM) in the brain slices prepared from WT mice. n.s., no significance.
7. The representative traces and statistical results showed that the amplitude of sEPSC in hippocampal neurons was decreased by incubating DIR (1 μM). ***, p < 0.001. n.s., no significance.
8. The long-term potentiation (LTP) of field EPSP recorded in the hippocampal CA1 region of the HO mice was apparently decreased, as compared with that of WT mice. ***, p < 0.001.
9. The decreased spent time and recognition index showed that the capacity of novel object recognition was reduced in the group treated with the DIR-Intron via the catheter implanted into the hippocampal CA1 region, delivering the DIR-Intron peptide locally through the subcutaneous minipumps (200 ng per mouse). O, old object; N, new object. **, p < 0.01.

Data shown are mean ± S.E.M. Two-tailed unpaired *t*-test or two-way ANOVA test followed by Bonferroni correction (h).


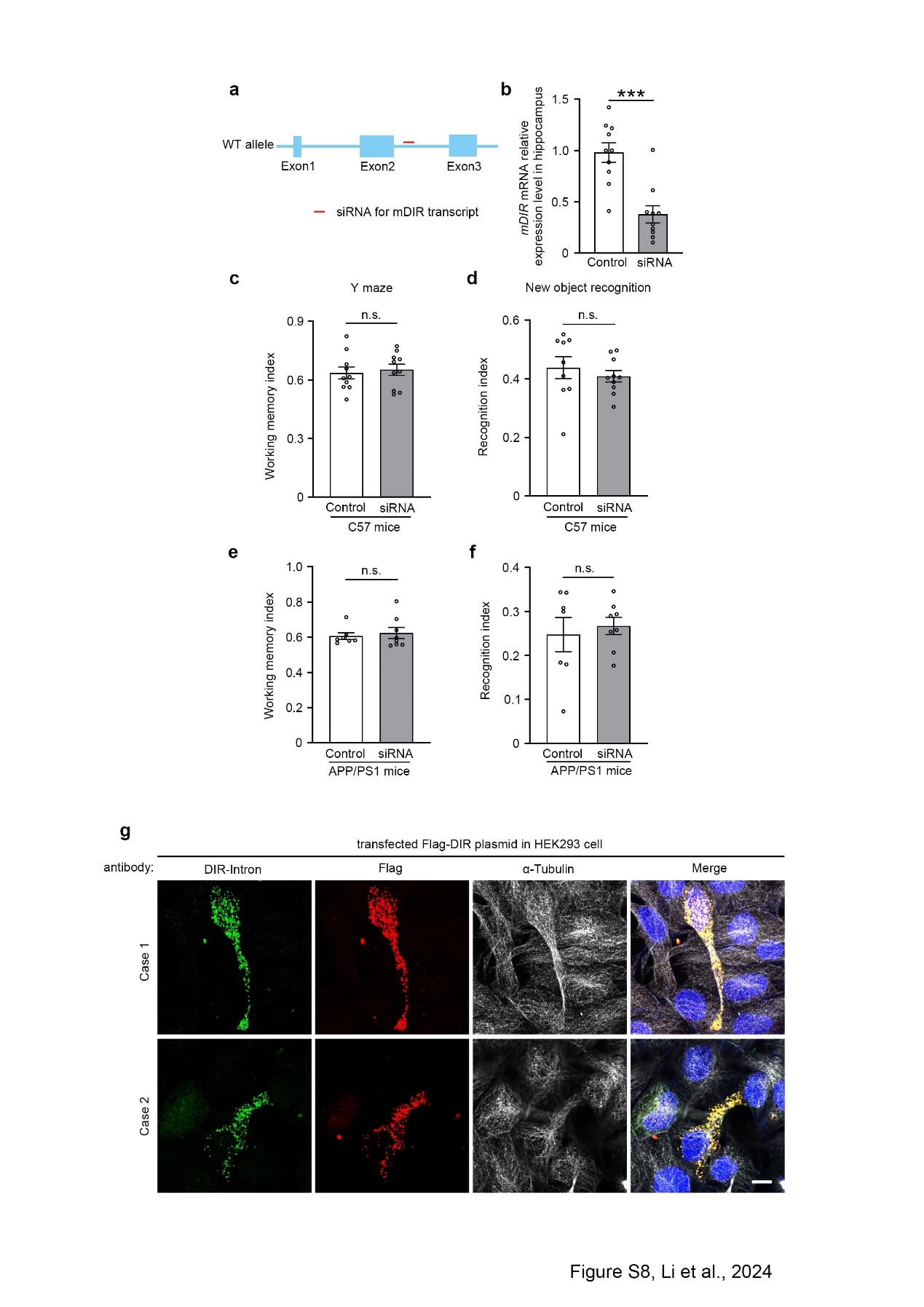


**Fig. S8, No effects of mDIR on working memory and novel object recognition**

1. Schematic diagram showed the action site of siRNA for mDIR transcript in the WT allele**.**
2. The statistical analysis showed that si*mDIR* apparently reduced mDIR mRNA level. ***, p < 0.001.
3. The Y maze alternative test showed that the working memory of si*mDIR* treated WT mice was not changed, compared with control groups of WT mice. n.s., no significance.
4. The capacity of novel object recognition of si*mDIR* treated groups had no change, compared with control groups of WT mice. n.s., no significance.
5. The Y maze alternative test showed that the si*mDIR* treatment could not change the working memory of APP/PS1mice, compared with the control groups of APP/PS1 mice. n.s., no significance.
6. The capacity of novel object recognition of si*mDIR* treated groups of APP/PS1 mice was not changed, as compared with the control groups of APP/PS1 mice. n.s., no significance.
7. In transfected the Flag-DIR plasmid in HEK293 cells, the DIR antibody (anti DIR-Intron) only stained in transfected cells (Flag-positive), not in non-transfected cells (Flag-negative), while α-Tubulin indicated the cell profile. Scale bars = 10 μm.

Data shown are mean ± S.E.M. Two-tailed unpaired *t*-test.


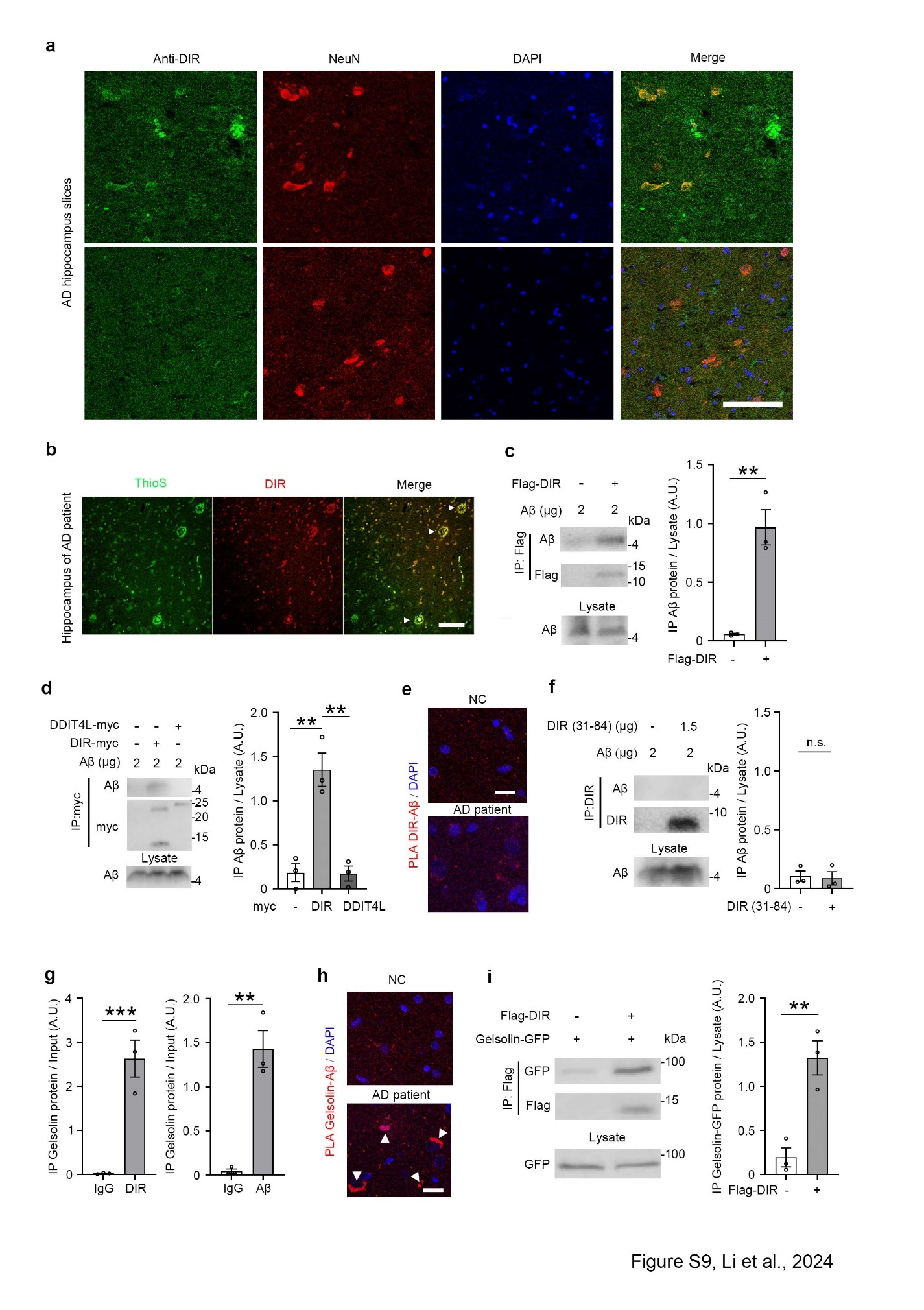


**Fig. S9, DIR expression in AD patients’ hippocampi, and DIR binding with gelsolin but not Aβ**

1. The antigen absorption of DIR antibody abolished the DIR-immunoreactive signals in the hippocampus slices of AD patients. Scale bar = 100 μm.
2. Immunostaining showed that DIR was present in the thioflavine S (ThioS)-positive plaque (arrows) in the hippocampus of AD patients. Scale bar = 50 μm.
3. Immunoblotting and quantificational analysis showed that Aβ was precipitated by Flag antibodies in the lysate of HEK293 cells transfected with the plasmid expressing Flag-DIR. **, p < 0.01.
4. Co-immunoprecipitation and statistical results showed that in the mixture of lysate of HEK293T cells co-transfected plasmids expressing myc-DIR or DDIT4L-myc with additions of synthetic Aβ42, Aβ was found in the proteins precipitated with myc antibodies in the lysate of cells expressing DIR-myc, but not that expressing DDIT4L-myc. **, p < 0.01.
5. The PLA assay showed that DIR could not directly bind to Aβ in AD patients’ hippocampus. Scale bar = 50 μm.
6. Co-IP and statistical analysis showing that DIR (31-84) and Aβ42 did not directly interact. n.s., no significance.
7. Quantificational analysis showing that gelsolin was precipitated by DIR and Aβ antibodies in the lysates of hippocampus from the DIR-KI mice. **, p < 0.01. ***, p < 0.001.
8. The PLA assay showed that gelsolin bound to Aβ (arrows) in AD patients’ hippocampus. Scale bar = 50 μm.
9. Co-IP and statistical results showed that in the lysate of HEK293T cells co-transfected with plasmids expressing Flag-DIR and gelsolin-GFP, gelsolin-GFP was found in the proteins precipitated with Flag antibodies. **, p < 0.01.

Data shown are mean ± S.E.M. Two-tailed unpaired *t*-test.


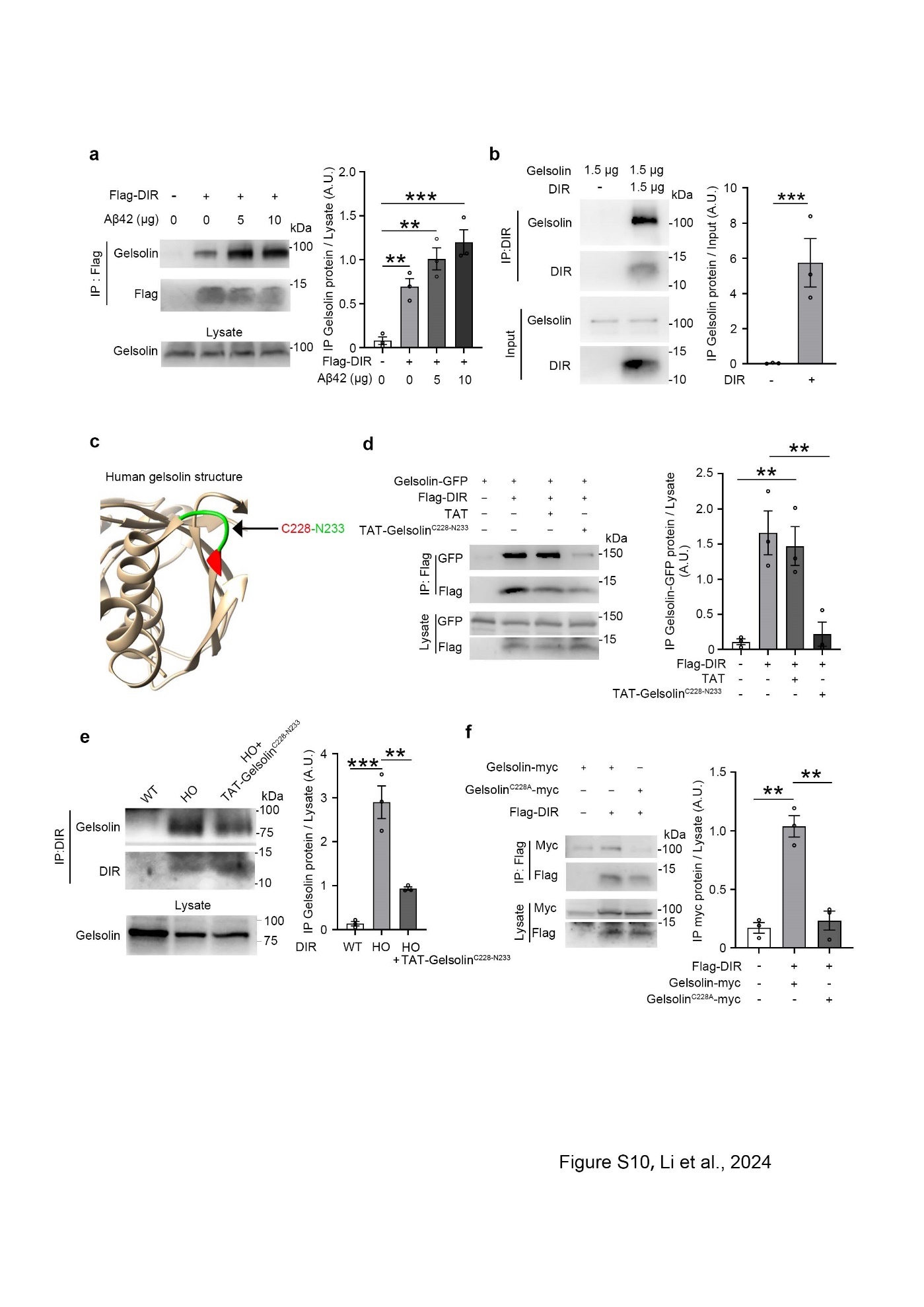


**Fig. S10, DIR-binding to the C228-N233 motif of gelsolin**

1. Co-IP and statistical results showing that in the lysate of HEK293T cells transfected with the plasmid expressing Flag-DIR with additions of different doses of synthetic Aβ42, gelsolin was found in the proteins precipitated with Flag antibodies, and increased binding level in the group with high doses of Aβ42 (5, 10 μg). **, p < 0.01. ***, p < 0.001.
2. Co-IP and statistical results showing that in the complex including purified protein of gelsolin and DIR, gelsolin was found in the proteins precipitated with DIR antibody. ***, p < 0.001.
3. By the combination of homology modelling and in silico docking, the C228-N233 motif in gelsolin (Gelsolin^C228-N233^) was predicted to be the docking site for DIR. C228 in gelsolin could be the key point for the interaction between DIR and gelsolin.
4. Co-IP and quantificational analysis showing that in the lysate of HEK293 cells co-transfected with plasmids expressing Flag-DIR and Gelsolin-GFP, the TAT-Gelsolin^C228-N233^ reduced the DIR/Gelsolin interaction. **, p < 0.01.
5. Co-IP and quantificational analysis showing that in the hippocampus tissue of WT and HO mice, gelsolin was found in the proteins precipitated with DIR antibody in the HO mice. The TAT-Gelsolin^C228-N233^ apparently reduced the DIR/Gelsolin interaction in the HO mice. **, p < 0.01. ***, p < 0.001.
6. Co-IP and quantificational analysis showing that in the lysate of HEK293 cells co-transfected with plasmids expressing Flag-DIR and Gelsolin-myc or Gelsolin^C228A^-myc, Gelsolin-myc, not Gelsolin^C228A^-myc, were found in the proteins precipitated with Flag antibodies. **, p < 0.01.

Data shown are mean ± S.E.M. Two-tailed unpaired *t*-test.


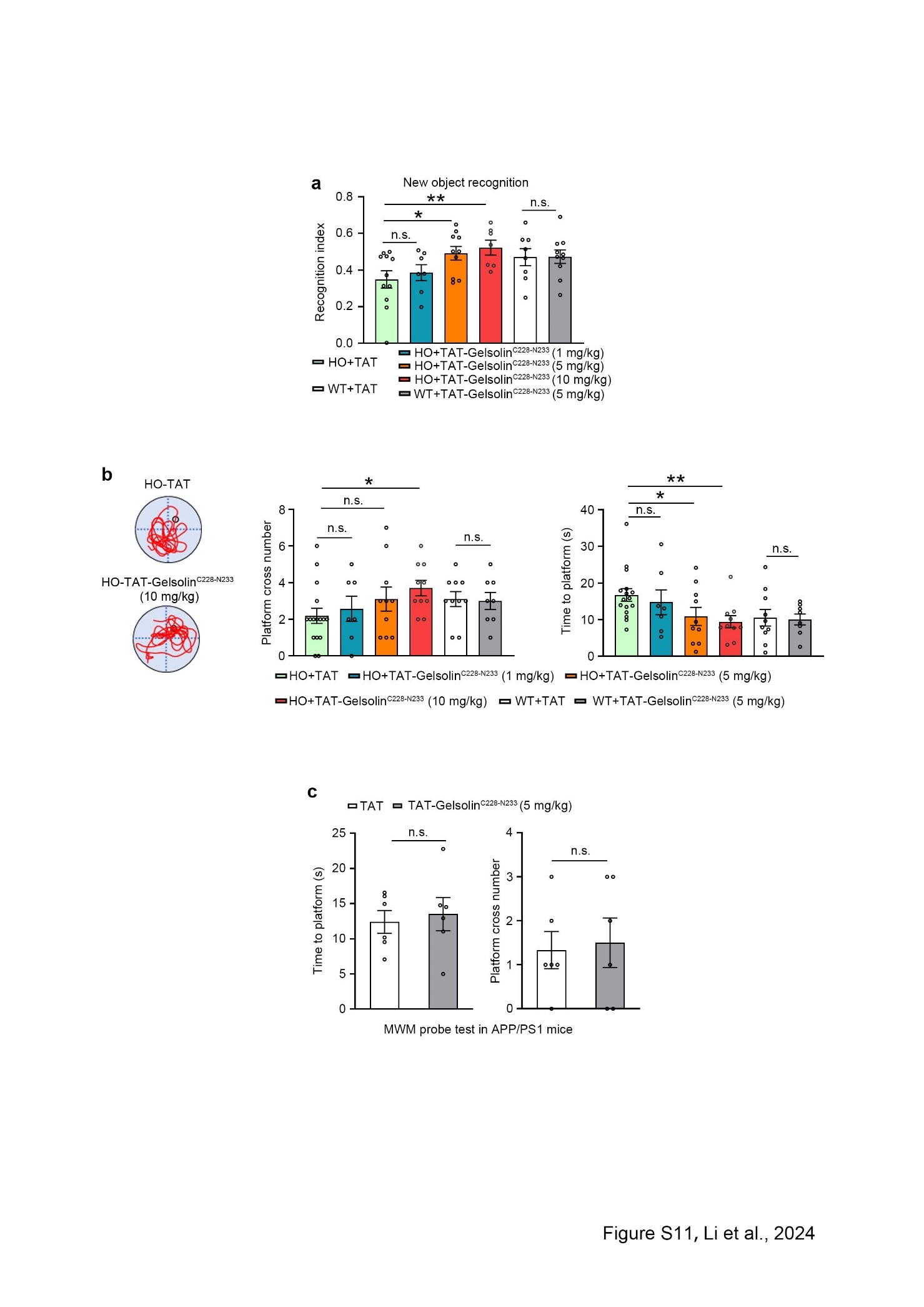


**Fig. S11, TAT-Gelsolin^C228-N233^-reversed learning and memory in the DIR-KI mice**

1. The capacity of novel object recognition was reversed by the TAT-Gelsolin^C228-N233^ treatment in a dose-dependent manner in the HO mice (1, 5, 10 mg/kg), as compared with the HO mice treated with TAT and WT mice treated with TAT or TAT-Gelsolin^C228-N233^. *, p < 0.05. **, p < 0.01. n.s., no significance.
2. Left, representative trial traces of individual mice on probe test day.  Right, statistical results showing that in the probe test to assess spatial memory, the mice treated with the TAT-Gelsolin^C228-N233^ increased time of crossing platform and spent less time to find platform in a dose-dependent manner (1, 5, 10 mg/kg), compared with the mice treated with TAT and WT mice treated with TAT or TAT-Gelsolin^C228-N233^. *, p < 0.05. **, p < 0.01. n.s., no significance.
3. The statistical results showing that in the probe test to assess spatial memory, the APP/PS1 mice treated with the TAT- Gelsolin^C228-N233^(5mg/kg) showed similar latency to reach platform and cross number in MWM test compared with the mice treated with TAT. n.s., no significance.

Data shown are mean ± S.E.M. Two-tailed unpaired *t*-test.


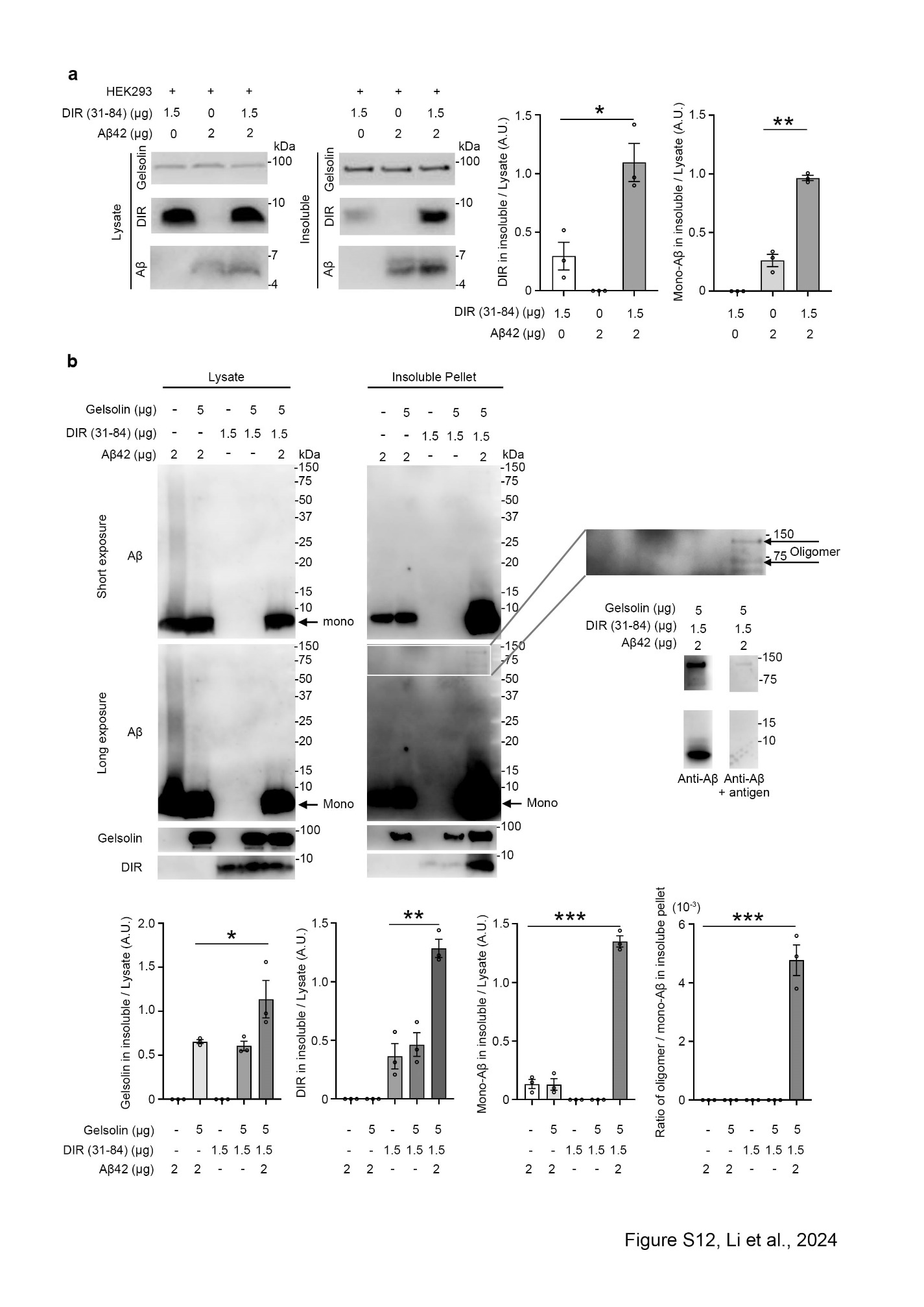


**Fig. S12, Enhanced insolubilization of DIR, Aβ and gelsolin**

1. The co-IP experiments and statistical results showing that in the mixture of lysate of HEK293T cells, which endogenously express gelsolin, with additions of synthetic Aβ42 or DIR (31-84), only both DIR (31-84) and Aβ42 existing could enhance the insolubilization of DIR and Aβ. *, p < 0.05. **, p < 0.01.
2. The co-IP experiments and statistical results showing that in the mixture of purified gelsolin protein with synthetic Aβ42 or DIR (31-84), only gelsolin, Aβ42 and DIR (31-84) concurrence increased Aβ42 (and form oligomer) or DIR (31-84) insolubilization. Immunoblotting showed Aβ oligomer immunoreactive bands (~130 and 70 kDa, arrowheads) detected with the antibody against Aβ42, but not by the pre-absorbed antibody in the mixture. *, p < 0.05. **, p < 0.01. ***, p < 0.001.

Data shown are mean ± S.E.M. Two-tailed unpaired *t*-test.

**
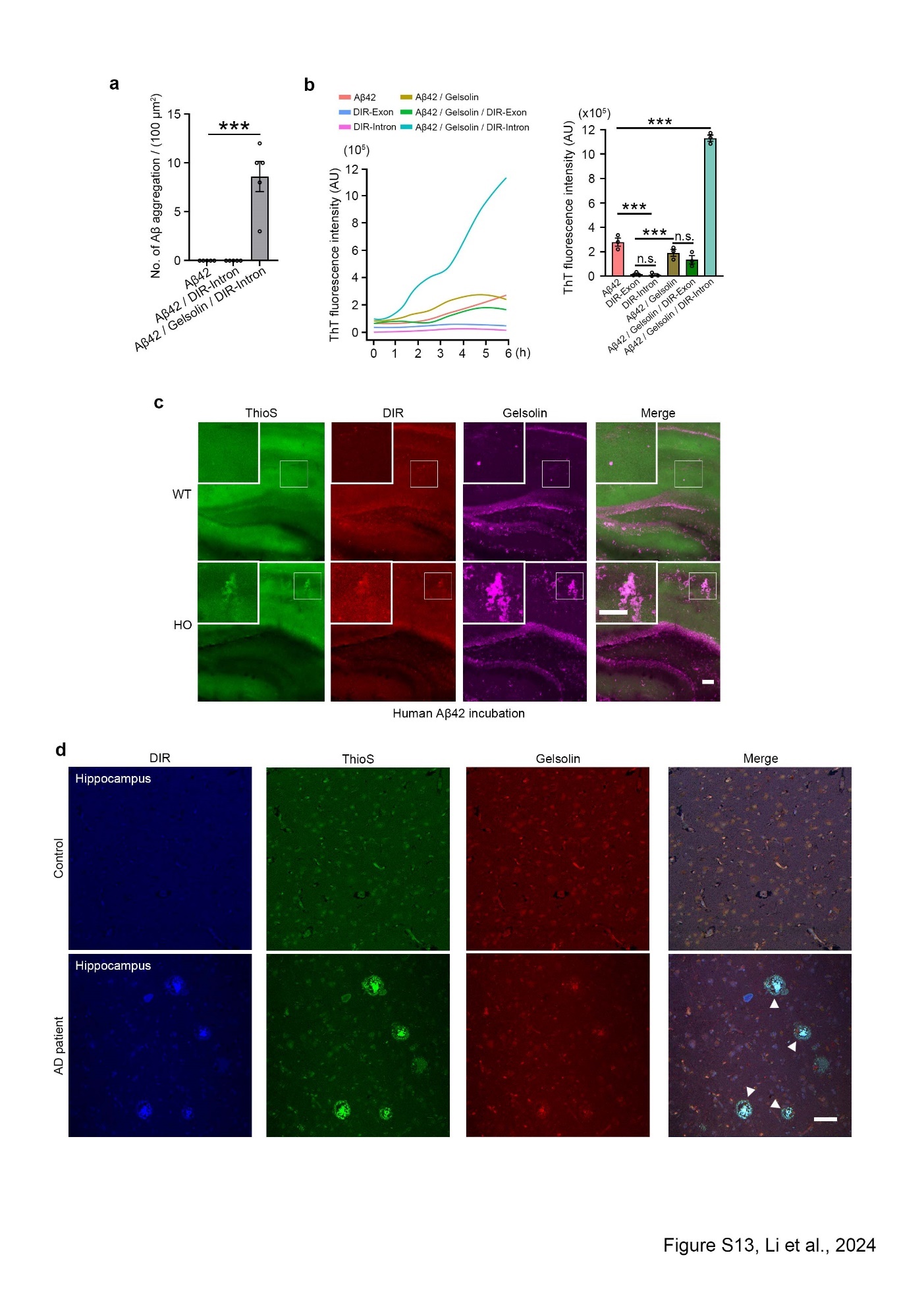
**

**Fig. S13, DIR-mediated Aβ plaque formation**

1. Quantificational analysis of electron microscope for Aβ42 (5 μM)/gelsolin (0.1 μM)/DIR-Intron (1 μM) incubation induced Aβ42 oligomerization. ***, p < 0.001.
2. The representative traces and statistical results of ThT fluorescence assay showing that the incubation of Aβ42, gelsolin and DIR-Intron increased the ThT fluorescence intensity. ***, p < 0.001. n.s., no significance.
3. The immunostaining showing that in the hippocampus slices of 6-month-old homogenous DIR-KI (HO) mice (n = 3), but not that of 6-month-old wildtype (WT) mice (n = 3), incubated with the synthetic human Aβ42 (100 μM) for 6 h, thioflavine S (ThioS)-positive plaque appeared and co-existed with DIR and gelsolin. Scale bars = 100 μm.
4. The immunostaining showing that in AD patients (n = 3), but not control individuals (n = 3), ThioS-positive dense-core plaque co-localized with DIR and gelsolin (arrows) in the hippocampal DG area. Scale bar = 50 μm. Quantitative analysis showed that DIR, gelsolin and ThioS-positive in 86.3% dense-core plaque in cerebral cortex, in 92.8% dense-core plaque in the hippocampus.

Data shown are mean ± S.E.M. Two-tailed unpaired *t*-test.

**
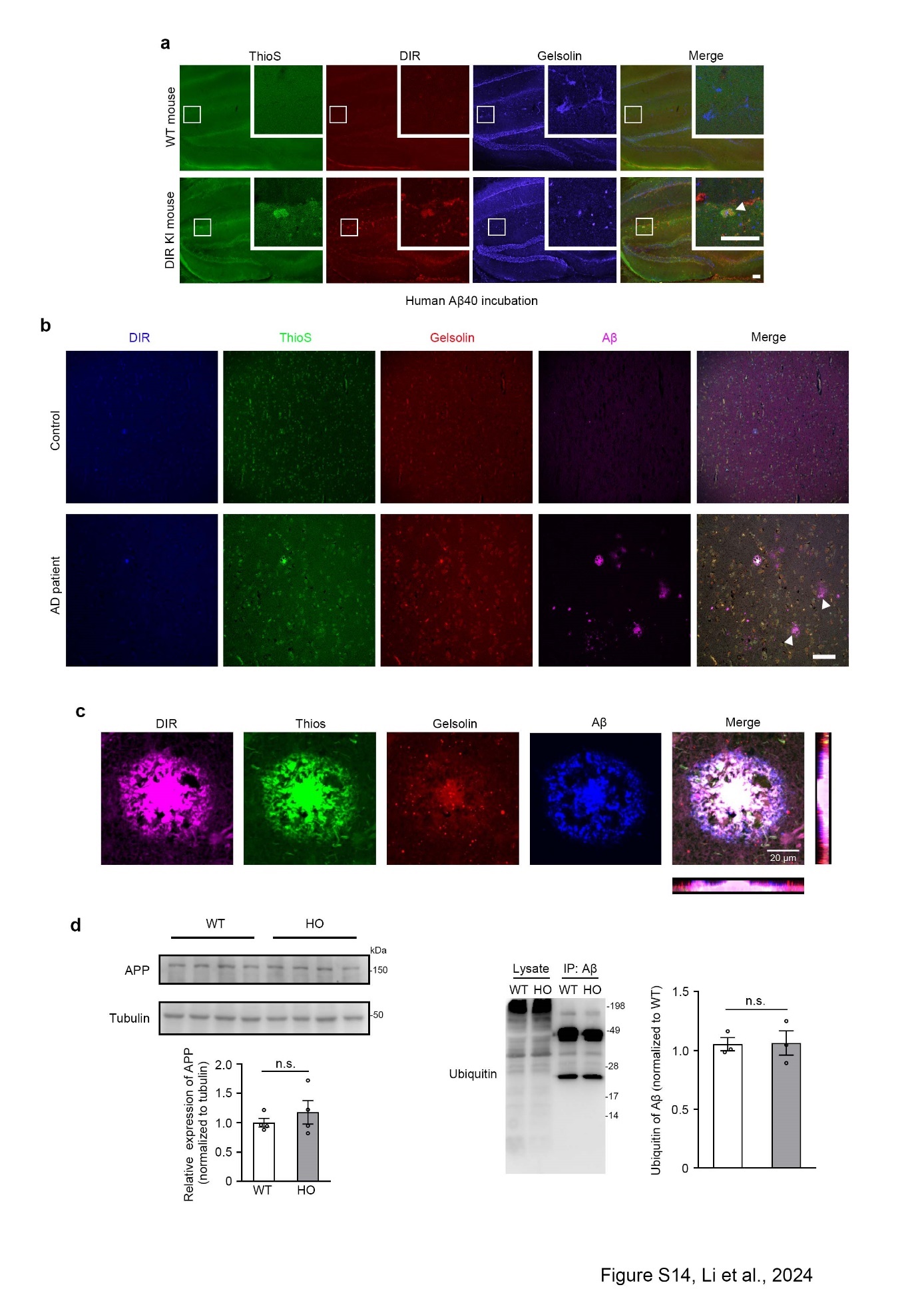
**

**Fig. S14, Colocalization of DIR, Aβ and gelsolin**

1. Immunostaining showed that in the hippocampal slices of the homogenous DIR-KI mice (n = 3), but not that of WT mice (n = 3), incubated with the synthetic human Aβ40 (100 μM) for 6 h, the thioflavine S-positive plaque (arrow) appeared and co-existed with DIR and gelsolin. Scale bars = 100 μm.
2. Immunostaining showed that in AD patients (n = 3), but not control individuals (n = 3), the Aβ-positive, thioflavine S-negative diffuse plaques (arrows) were not co-localized with DIR and gelsolin in the hippocampal DG area. Scale bar = 50 μm.
3. The confocal image of DIR, Aβ, gelsolin and ThioS in hippocampus of AD patients. Scale bar = 20 μm.
4. Immunoblotting and statistical results showed that the APP protein level and Aβ ubiquitin level were not changed in the hippocampus of HO mice. n.s., no significance.

Data shown are mean ± S.E.M. Two-tailed unpaired *t*-test.


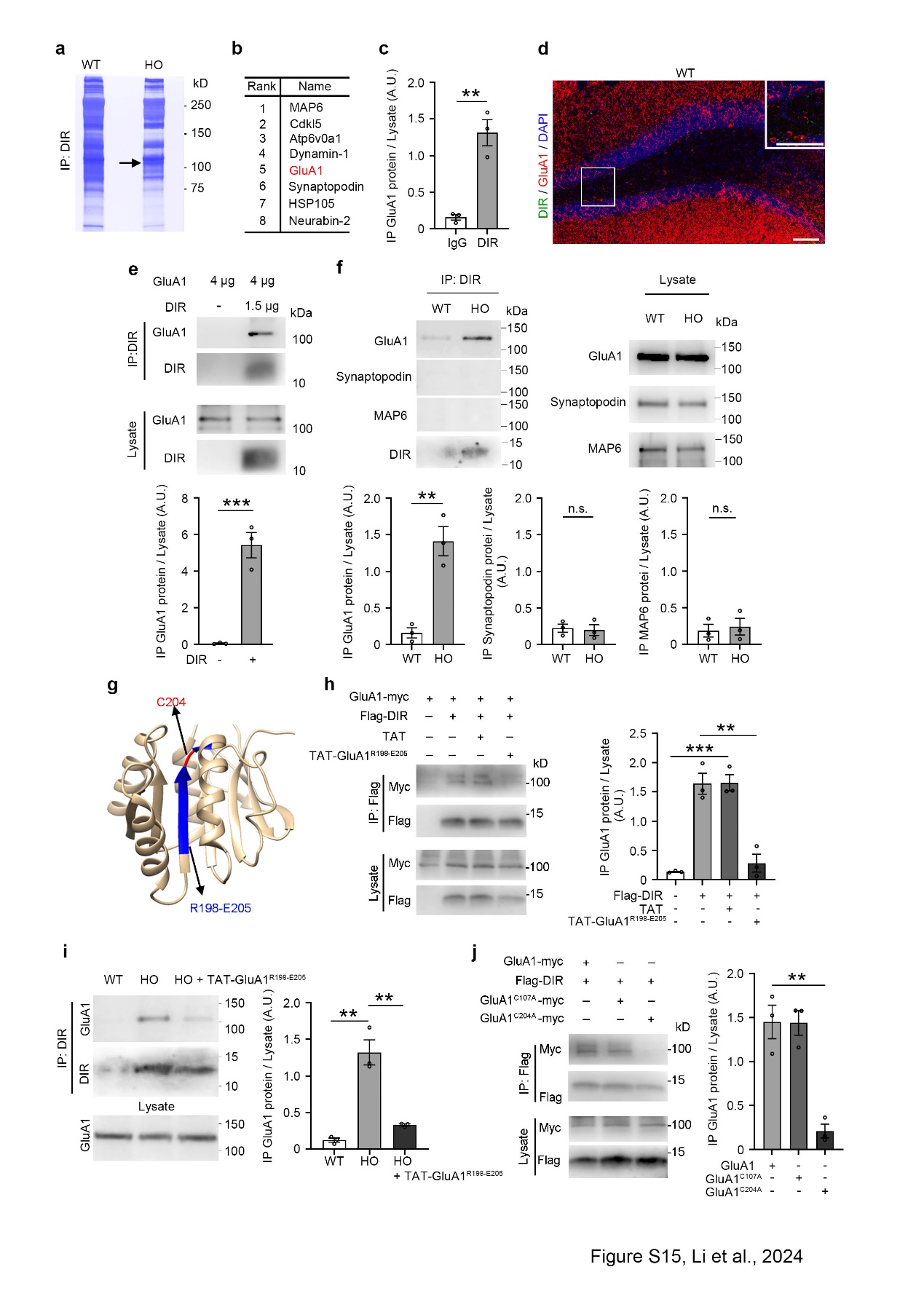


**Fig. S15, TAT-GluA1^R198-E205^-induced reduction in the DIR and GluA1 interaction**

1. Coomassie blue staining showed a band of molecular weight ~110 kDa in the DIR-antibody-precipitated proteins from the hippocampus of homogenous DIR-KI (HO) mice, but not wildtype (WT) (n = 3).
2. The mass spectrometry identified top 8 molecules from the ~110 kDa band. GluA1 was in the list.
3. Quantificational analysis showing that GluA1 was precipitated by the DIR antibody in the lysate of hippocampus from the HO mice. **, p < 0.01.
4. The immunostaining showed that DIR was not stained and colocalized with GluA1-positive neurons in the WT mice. Scale bars = 50 μm.
5. Co-IP and statistical result showed that in the complex of purified GluA1 and DIR, GluA1 was found in the proteins precipitated with DIR antibody. ***, p < 0.001.
6. Co-IP and statistical result showed that in the hippocampus tissue of WT and HO mice, GluA1, neither MAP6 nor synaptopodin, was markedly increased in the proteins precipitated with DIR antibody in the HO mice. **, p < 0.01. n.s., no significance.
7. By the combination of homology modelling and in silico docking, the R198-E205 motif in GluA1 (GluA1^R198-E205^) was predicted to be the docking site for DIR. C204 in GluA1 could be the key point for the interaction between DIR and GluA1.
8. Co-IP and statistical result showed that in the lysate of HEK293 cells co-transfected with plasmids expressing Flag-DIR and GluA1-myc, GluA1-myc was found in the proteins precipitated with Flag antibodies. TAT-GluA1^R198-E205^ apparently reduced the DIR/GluA1 interaction. **, p < 0.01. ***, p < 0.001.
9. Co-IP and statistical result showed that in the hippocampus tissue, GluA1 was found in the proteins precipitated with DIR antibody in the HO mice. The TAT-GluA1^R198-E205^ apparently reduced the DIR/GluA1 interaction in the HO mice. **, p < 0.01.
10. Co-IP and statistical result showed that in the lysate of HEK293 cells co-transfected with plasmids expressing Flag-DIR and GluA1-myc or GluA1^C107A^-myc or GluA1^C204A^-myc, GluA1-myc and GluA1^C107A^-myc, not GluA1^C204A^-myc, were found in the proteins precipitated with Flag antibodies. **, p < 0.01.

Data shown are mean ± S.E.M. Two-tailed unpaired *t*-test.


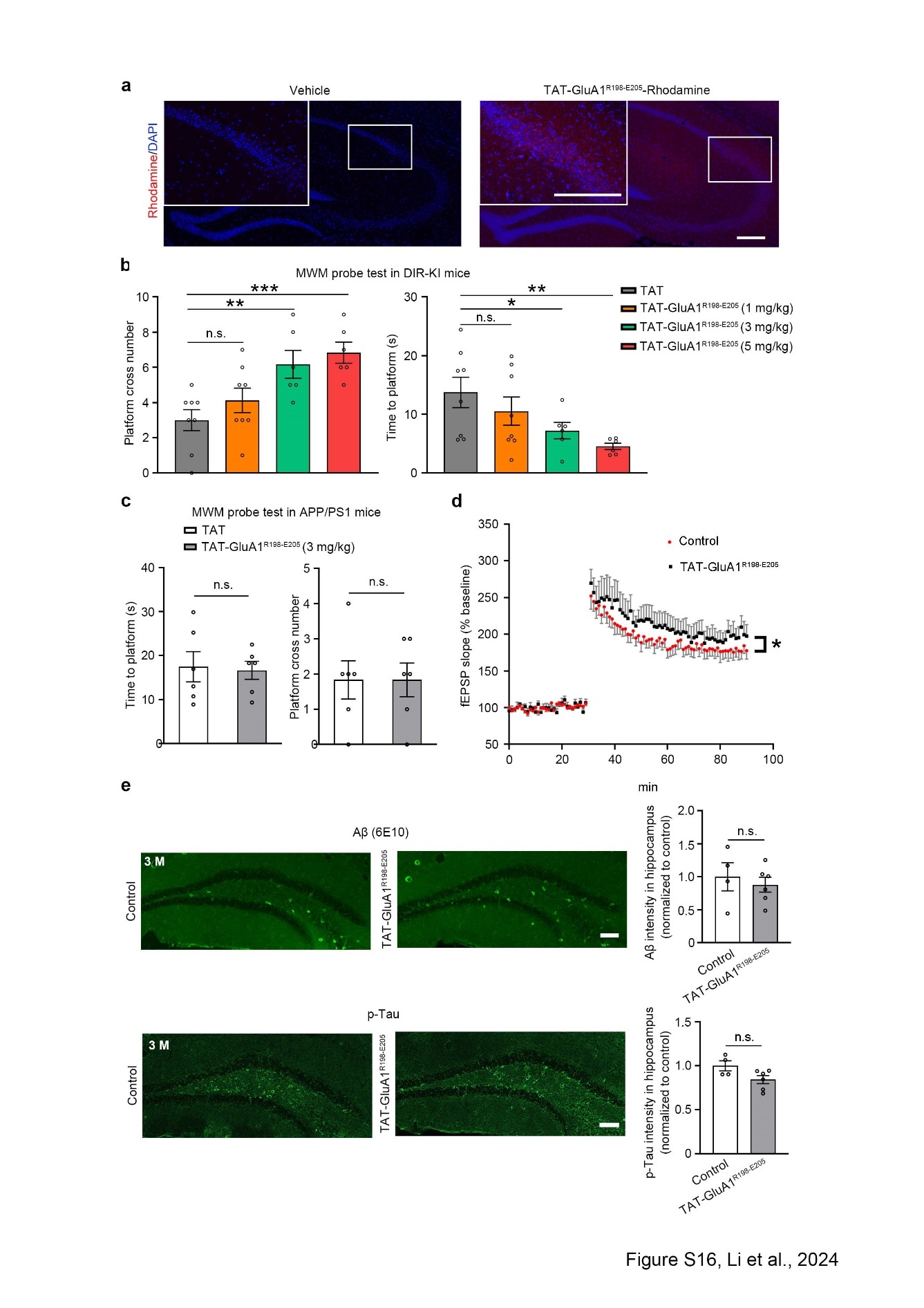


**Fig. S16, Enhanced learning and memory in the DIR-KI mice treated with TAT-GluA1^R198-E205^**

1. Immunostaining showed that TAT-GluA1^R198-E205^ (3 mg/kg, i.v.) labeled with rhodamine could cross the blood-brain barrier into the hippocampus (n=3). Scale bars = 100 μm.
2. The statistical results showing that in the probe test to assess spatial memory, the HO mice treated with TAT did not show a preference for the target quadrant, whereas the mice treated with the TAT-GluA1^R198-E205^ had more times for crossing platform and spent less time to find platform in a dose-dependent manner (1 mg/kg, 3 mg/kg, 5 mg/kg). *, p < 0.05. **, p < 0.01. ***, p < 0.001. n.s., no significance.
3. The statistical results showing that in the probe test to assess spatial memory, the APP/PS1 mice treated with the TAT- GluA1^R198-E205^ (3 mg/kg) showed similar latency to reach platform and cross number in MWM test compared with the mice treated with TAT. n.s., no significance.
4. The hippocampal LTP in the homozygous DIR-KI mice was apparently increased after the TAT-GluA1^R198-E205^ treatment, as compared with TAT treatment (n = 5 mice per group). *, p < 0.05.
5. Immunostaining and statistical results showed that the Aβ and p-Tau expression in the hippocampal dentate gyrus in the 3-month-old homozygous DIR-KI mice was not changed after the TAT-GluA1^R198-E205^ treatment. Scale bars = 100 μm.

Data shown are mean ± S.E.M. Two-tailed unpaired *t*-test or two-way ANOVA test followed by Bonferroni correction (d).

**
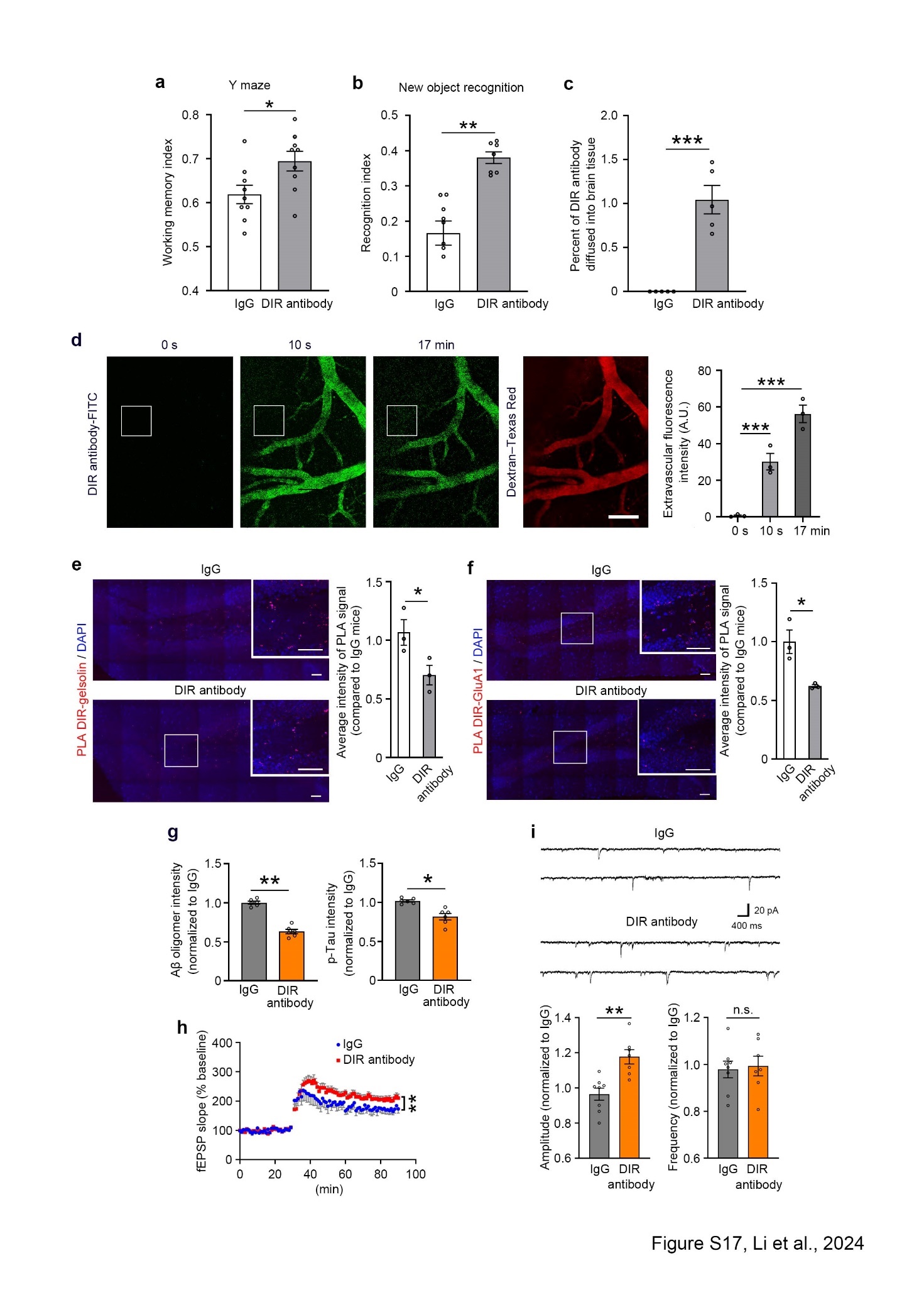
**

**Fig. S17, DIR mAb diffusion into the brain tissues in DIR-KI mice, and the reduction of DIR binding to GluA1 or gelsolin in the hippocampi of DIR-KI mice treated with DIR mAb**

1. The Y maze alternative test showed that the working memory could be partially reversed by the DIR mAb treatment in the DIR-KI mice, as compared with the mice treated with IgG. *, p < 0.05.
2. The capacity of novel object recognition was partially reversed by the DIR mAb treatment in the DIR-KI mice, as compared with the mice treated with IgG. **, p < 0.01.
3. The DIR antibody by intraperitoneal injection could cross the blood brain barrier (BBB) after 24 h by ELISA. ***, p < 0.001.
4. Two-photon imaging and statistical results of the mouse brain tissue after intravenous injection of FITC conjugated DIR antibody showed the capacity to cross the blood brain barrier. Dextran-Texas Red showed that FITC-tagged DIR antibody diffused from blood vessels into the brain tissue (n = 3 mice). ***, p < 0.001.
5. The PLA assay and statistical result showed that DIR antibody treatment could reduce DIR bound to gelsolin in the hippocampus of HO mice. Scale bars = 50 μm. *, p < 0.05.
6. The PLA assay and statistical result showed that DIR antibody treatment could reduce DIR bound to GluA1 in the hippocampus of HO mice. Scale bars = 50 μm. *, p < 0.05.
7. Quantificational analysis of expression for Aβ- and p-Tau in the hippocampal dentate gyrus in the 6-month-old HO mice treated with the DIR mAb. *, p < 0.05. **, p < 0.01.
8. The LTP in the HO mice was apparently increased after the DIR mAb treatment, as compared with IgG treatment (n = 5 mice per group). **, p < 0.01.
9. The representative traces and statistical results showed that the spontaneous excitatory postsynaptic current (sEPSC) of hippocampal neurons was increased after the DIR mAb treatment, as compared with IgG treatment. The amplitude, not frequency of sEPSC in hippocampal neurons was apparently increased after the DIR mAb treatment, as compared with IgG treatment. **, p < 0.01. n.s., no significance.

Data shown are mean ± S.E.M. Two-tailed unpaired *t*-test (a, b, c, d, e, f, g, i) or two-way ANOVA test followed by Bonferroni correction (h).

**Supplementary Table 1: DDIT4L in different species**

Human DDIT4L: MVATGSLSSKNPASISELLDCGYHPESLLSDFDYWDYVVPEPNLNEVIFEESTCQNLVKMLENCLSKSKQTKLGCSKVLVPEKLTQRIAQDVLRLSSTEPCGLRGCVMHVNLEIENVCKKLDRIVCDSSVVPTFELTLVFKQENCSWTSFRDFFFSRGRFSSGFRRTLILSSGFRLVKKKLYSLIGTTVIEGS*

Chimpanzee DDIT4L: MVATGSLSSKNPASISELLDCGYHPESLLSDFDYWDYVVPEPNLNEVIFEESTCQNLVKMLENCLSKSKQTKLGCSKVLVPEKLTQRIAQDVLRLSSTEPCGLRGCVMHVNLEIENVCKKLDRIVCDSSVVPTFELTLVFKQENCSWTSFRDFFFSKGRFSSGFRRTLILSSGFRLVKKKLYSLIGTTVIEGS*

Mouse DDIT4L: MVATGSLSSKNPASISELLDGGYHPGSLLSDFDYWDYVVPEPNLNEVVFEETTCQNLVKMLENCLSRSKQTKLGCSKVLVPEKLTQRIAQDVLRLSSTEPCGLRGCVMHVNLEIENVCKKLDRIVCDASVVPTFELTLVFKQESCPWTSLKDFFFSRGRFSSGLKRTLILSSGFRLVKKKLYSLIGTTVIEEC*

**Supplementary Table 2: Clinical information**

| ID | Hospital ID | Age | Gender | Pathological diagnosis |
| --- | --- | --- | --- | --- |
| A7098 | 42717098 | 61 | Female | AD |
| A8750 | 42788750 | 60 | Male | AD |
| A1844 | 42781844 | 83 | Female | AD |
| A0398 | 42710398 | 57 | Female | AD |
| A2205 | 42702205 | 54 | Female | AD |
| A2669 | 42752669 | 73 | Female | AD |
| A5125 | 42005125 | 64 | Male | AD |
| N0781 | 42740781 | 73 | Male | NC |
| N5753 | 42705753 | 71 | Female | NC |
